# Dynamic response of Blue Honeysuckle fruit-stem system based on mathematical model

**DOI:** 10.1371/journal.pone.0344122

**Published:** 2026-03-04

**Authors:** Yuan Wei, Wang Ruiyin, Wang Yecheng, Feng Fang, Ma Decai

**Affiliations:** 1 College of Engineering, Northeast Agricultural University, Harbin, China; 2 College of Arts and Sciences, Northeast Agricultural University, Harbin, China; 3 Sino-French Institute of Nuclear Engineering & Technology, Sun Yat-sen University, Zhuhai, China; China University of Mining and Technology, CHINA

## Abstract

To investigate the motion and detachment behavior of Blue Honeysuckle fruit during vibratory harvesting, the fruit-bearing branch was modeled as a constant strength beam, and an analytical series solution for its dynamic response was derived. This solution shows that the branch undergoes approximately simple harmonic motion under low frequency excitation. Subsequently, a dynamic model of the fruit-stem system subject to branch oscillation with the fruit stem at an angle to the excitation direction was developed. When the fruit stem is perpendicular to the excitation direction, the stress within it can exceed its allowable limit under low frequency excitation, leading to fruit detachment. Conversely, when the fruit stem is aligned parallel to the excitation direction, fruit detachment results from the vibrational instability of the fruit-stem system. For the case where a small angle exists between the stem and the excitation direction, the criterion for vibrational instability remains consistent with that of the parallel scenario. Modal analysis and frequency sweep analysis were performed using the finite element method (FEM) to study the vibration response characteristics of the fruit-stem system. The finite element simulation results are in close agreement with theoretical calculations. Field vibration experiments were carried out in an experimental plot. The experimental data confirmed that the simplified dynamic model can accurately predict the fruit detachment patterns. The findings of this study can serve as a theoretical foundation for the design, selection, and optimization of mechanical harvesting equipment for berry crops.

## 1. Introduction

The harvesting of Blue Honeysuckle (*Lonicera caerulea* L.) is characterized by significant seasonality and high labor intensity, with canopy-shaking harvesting systems becoming a major research focus due to their high efficiency [1]. Vibratory harvesting is a technique where mechanical shakers are employed to impart vibrations to the tree, leading to the detachment of mature fruits [2,3]. However, Blue Honeysuckle berries are fragile and highly prone to damage during mechanical harvesting. Moreover, due to inherent biological variability among individual plants, experimental parameters often show significant variability. The vibration process can also cause excessive stress and damage to trees. Therefore, investigating the dynamic response of fruits to vibrational excitation is crucial for optimizing harvesting efficiency, reducing fruits damage, and minimizing mechanical injury to the tree structure.

Many vibration models have been established to describe fruit detachment during vibratory harvesting. The classical fruit vibration model is the double-compound pendulum model [4], which simplifies the fruit-stem system into a three-degree-of-freedom model and derives nonhomogeneous Hill’s equations of motion, which can predict the frequency conditions for fruit detachment, with or without the stem. Rand and Cooke [5] pointed out that the frequency of the in-phase mode (where the fruit and stem swing as a rigid body) is almost independent of amplitude, while the frequency of the out-of-phase mode (where the fruit and stem swing in opposite directions) decreases significantly with increasing amplitude. Tsatsarelis [6] established a two-degree-of-freedom model for a fruit-stem system with linear viscous damping and nonlinear air resistance as external damping and investigated the effect of vibration on olive fruit detachment. Wang et al [7] established the dynamic equations for Camellia Oleifera fruit during vibratory harvesting and derived the vibration response and the detachment inertial force. Yan et al [8] developed a planar double pendulum dynamic model of the grape, from which the theoretical angular velocity of berry oscillation was derived. Xu et al [9] established a vibration-collision coupling analysis method for grape clusters and proposed a minimal vibration system. Zhou et al [10] studied and simulated the dynamic response of Jujube fruits, their results indicated that the location of the maximum stress on the fruit is related to the vibration direction.

The growing environment of fruit trees is inherently complex. Many scholars have established dynamic models of fruit trees through theoretical modeling, numerical analysis, and experimental modal analysis. Mei et al [11] investigated the vibratory harvesting principles of wolfberry branches and established a mechanical model for vibratory harvesting based on a simplified cantilever beam model. Alper et al [12] studied the transmission of vibration from the point of force application through the structure of an orange tree to the fruit attachment node. Láng [13] built a simple tree structure model composed of a trunk and main roots. This model included mass, spring, and damping elements, all reduced to the external end of the main roots. Bu et al [14] developed a finite element model of the branch-stem-fruit system, employing an orthotropic material model and a cohesive zone model to represent the mechanical properties of the branch and the cohesive zone, respectively. Wu et al [15] established mathematical models for fruits with short and long stems, demonstrating that the inertial force generated by fruit vibration plays a crucial role in fruit separation.

Numerous scholars have used numerical simulations and experiments to determine the optimal harvesting parameters for aspects such as harvesting efficiency and fruit damage ratio. Ortiz et al [16] reported that the use of an experimental shaker resulted in a higher ratio of fruits with minor injuries. Zhou et al [17] investigated the high damage ratio during the mechanical harvesting of fresh sweet cherries by extracting parameters and the number of fruit-limb collisions from high-speed videos to determine factors affecting fruit harvest and damage. He et al [18,19] evaluated fruit harvesting efficiency using different excitation positions and demonstrated that harvesting efficiency increased at positions farther from the excitation point.

While numerous studies have been conducted on vibratory harvesting, research on the underlying mechanisms remains relatively limited [20,21]. Since the orientation of the stem is not always vertically downward, the influence of stem orientation on fruit detachment has rarely been incorporated into theoretical models. The angular difference between the stem orientation and the excitation direction can lead to varying harvesting outcomes. In many harvesting experiments, the common practice of selecting and analyzing only a subset of parameters to obtain seemingly optimized results requires further scrutiny.

Although numerous studies have established vibration models for fruit detachment during vibratory harvesting, most models assume a vertical orientation of the fruit stem, thereby failing to account for the diversity of stem orientations encountered under field conditions and their complex influence on detachment mechanisms. In particular, the influence of the angle between the stem and the excitation direction on detachment behavior remains underexplored.

To address these limitations, this study presents a dynamic model that explicitly incorporates the stem orientation relative to the excitation direction to elucidate the detachment mechanisms under varying stem angles. A constant strength beam model is employed to establish the geometric model of a Blue Honeysuckle branch. The analysis demonstrates that the steady-state response of the branch under excitation from a harvesting finger can be simplified as a harmonic vibration. Based on the assumption of a specific angle between the fruit stem and the excitation direction, the governing dynamic equations are derived, and the steady-state solutions are analyzed to illustrate the vibration response of the stem and the force exerted on it under different excitation directions. Additionally, a finite element model is established to obtain the vibration amplitude and the von Mises stress of the stem at different excitation frequencies. Finally, experimental tests are conducted to validate the proposed theoretical model.

## 2. Materials and methods

### 2.1. Materials

**Fig 1:**
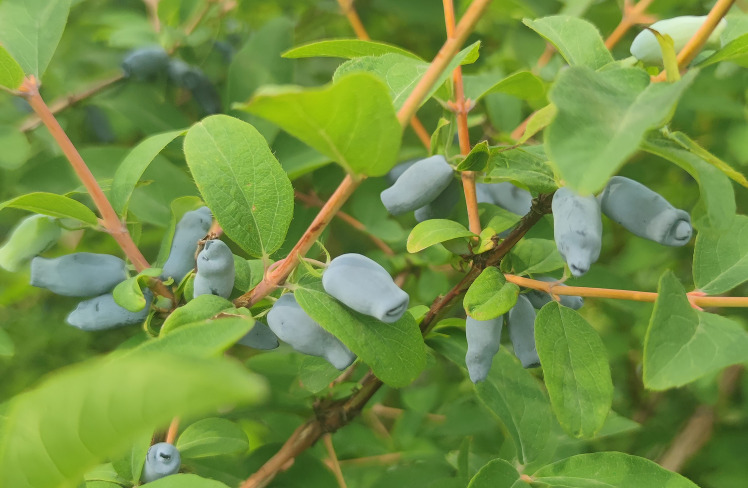
Blue Honeysuckle. Note: This is an original figure created by the authors.

The experiments were conducted in June, 2025, at the Horticultural Experiment Station of Northeast Agricultural University in Heilongjiang Province, China, using healthy and vigorous Blue Honeysuckle plants (*Lonicera caerulea* L.; 5–8 years old). The experiment commenced when the fruits transitioned from red to purple, as illustrated in Fig 1.

The key dimensions of the fruits were measured using a digital caliper. The mass of the fruits and branches were determined using an electronic balance, and the tensile strength of the fruit stems was evaluated using a universal testing machine. The obtained parameters are presented in Table 1. Tensile tests were conducted using an ST series universal testing machine, specifically a 1ST single-column model (Model: 1ST-50ST, Tinius Olsen Inc., USA) with a speed precision of ±0.05% and a load capacity range of 0–1 KN. A force transducer was integrated with the testing system to perform tensile tests on the fruit branch. The mass of the fruits and stems was measured using a high-precision electronic balance (Model: AH-A-1002G, resolution: 0.01 g). Diameter measurements were taken with a digital caliper (Model: CJW888, resolution: 0.02 mm). An eccentric link-slide mechanism developed by our research group was employed as the vibrating actuator. The rotational speed of the eccentric mechanism was controlled by a variable frequency drive (Model: DELTA VFD007M43B, frequency range: 0–60 Hz) that adjusted the motor speed. The rotational speed of the eccentric wheel was measured using a non-contact laser tachometer (Model: SW-6234C, effective measuring distance: 50–200 mm, accuracy: ±(0.05% of reading + 1 digit)). The vibration amplitude was adjusted by altering the position of the connecting rod on the eccentric wheel.

**Table 1:** Basic parameters of Blue Honeysuckle tree.

Age (year)	5-8
Density of the fruit branch ρ ($\text{kg}\cdot {\text{m}}^{-\text{3}}$)	887±46
Elastic modulus of the fruit branch E (Mpa)	5023±643
Allowable stress of the fruit branch [σ] (Mpa)	35.3±13.7
Length of the fruit branch (mm)	885±171
Basal diameter of the fruit branch (mm)	9.3±2.4
Apical diameter of the fruit branch (mm)	2.9±0.6
Fruit stem length l (mm)	7.3±2.1
Diameter of the de-corticated fruit stem d (mm)	0.53±0.13
Mean fruit weight m (g)	0.86±0.26
Fruit length (mm)	18.21±5.53
Fruit width (mm)	7.68±2.45
Bulk density of fruit ($\text{kg}\cdot {\text{m}}^{-\text{3}}$)	1280±167

Values presented are mean ± standard deviation.

### 2.2. Method

Vibratory harvesting involves imparting impact to the fruit trees, which generates excitation forces that cause the fruit-stem system to vibrate. The fruit detaches when the stem fractures. The vibration mechanism is an eccentric linkage mechanism. The rotational speed of the eccentric linkage mechanism is adjusted by a frequency converter that controls the motor speed, and the rotational speed is measured by a tachometer. The vibration amplitude is adjusted by altering the eccentricity through changing the mounting position of the linkage on the eccentric wheel. The mass of the harvested fruits is measured using an electronic balance.

The rotational speed was tested at 300, 400, 500, 600, and $\text{700r}\cdot {\text{min}}^{-1}$ (31.42, 41.87, 52.33, 62.83, and $\text{73.27rad}\cdot {\text{s}}^{-1}$). The harvesting finger excites the fruit branches to oscillate in a reciprocating manner. The vibration amplitude was maintained at 39mm and 53mm, and the spacing between the harvesting fingers was fixed at 40mm. he excitation duration was set at 4s. A total of 10 test groups were conducted, with each test repeated 3 times. The mean values of the harvest efficiency and damage ratio from the three replicates were used as the final data. Under identical experimental conditions except for an amplitude of 39mm, five sets of trials were conducted on fruit trees where fruits were manually removed and whose orientation deviated from the vertical direction by an angle less than π/4. Each set of trials was repeated three times, and the average values of the harvesting efficiency and damage ratio from the three repetitions were taken as the experimental results. As a comparative experiment, five sets of trials were conducted on fruit trees where fruits were manually removed and whose orientation deviated from the horizontal direction by an angle less than π/4. Each set was likewise repeated three times, with the average harvesting efficiency and damage rate calculated as the final data.

#### 2.2.1. Fruit branch morphology.

The morphology of a fruit branch significantly influences its vibrational characteristics. For instance, J. Liu et al [22] systematically altered the architecture of *Camellia oleifera* trees through pruning to evaluate the vibrational response and attenuation of canopies with distinct branch angles. In reality, branch section geometry arises from the interplay of multiple biological and physical factors, encompassing plant growth strategies, environmental adaptation, structural mechanics, and resource allocation. For Blue Honeysuckle, it is hypothesized that branches develop in a configuration approximating that of a beam of uniform strength. Based on this hypothesis, the overall morphology of a fruit branch can be derived.

**Fig 2:**
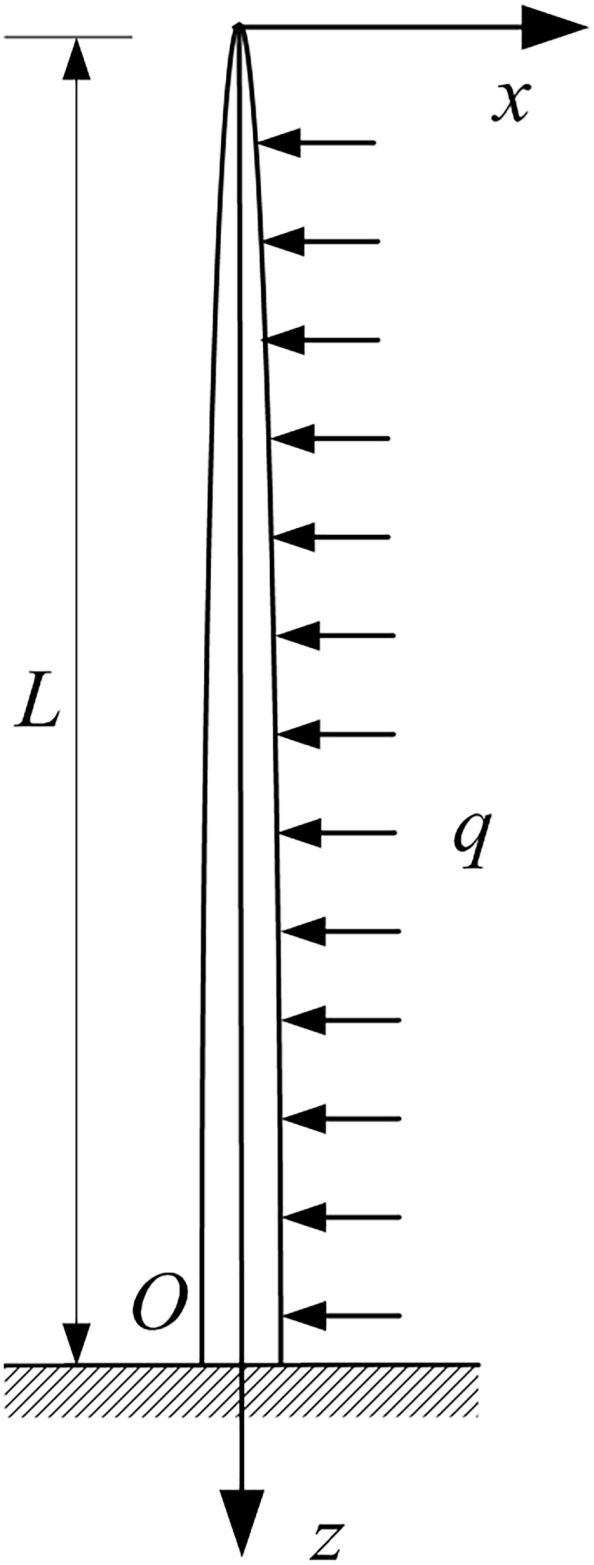
Fruit branch under uniformly distributed load.

Consider a uniform strength branch modeled as a cantilever beam of length $z_{\text{1}}$ with a circular cross-section. This beam is subjected to a uniformly distributed load q. The coordinate origin is placed at the beam tip, and the structure is illustrated in Fig 2.

The bending moment at a distance z from the branch tip is:


$$M_{s}\left(z\right)=\frac{qz^{\text{2}}}{\text{2}}$$ (1)

Assuming that under a distributed load q, the maximum bending stress $\sigma_{\text{max}}$ at any cross section of the branch remains constant, and applying the formula for maximum bending stress in a circular cross-section:


$$\sigma_{\text{max}}=\frac{32M_{s}\left(z\right)}{\pi d(z{)}^{\text{3}}}$$ (2)

By substituting the bending moment expression from Eq (1) into the Eq (2), the diameter distribution function of the fruit branch is derived as:


$$d\left(z\right)={\left(\frac{16q}{\pi \sigma_{\text{max}}}\right)}^{1/3}z^{2/3}$$ (3)

#### 2.2.2. Dynamic model of fruit branch.

When the harvesting finger strikes the branches at a specific frequency, the vibration output response of the branch is $f_{F}\left(z,t\right)$, and it can be expressed as:


$$f_{F}\left(z,t\right)=f_{d}\left(z,t\right)+f\left(z,t\right)$$ (4)

where $f_{d}\left(z,t\right)$ is the transient response of the branch, and f(z,t) is the steady-state response of the branch. By modeling the fruit branch as a cantilever beam, the differential equation governing its bending vibration is derived as:


$$\frac{\partial^{\text{2}}}{\partial z^{\text{2}}}\left[EI_{b}\left(z\right)\frac{\partial^{\text{2}}f_{F}\left(z,t\right)}{\partial z^{\text{2}}}\right]+\rho A\left(z\right)\frac{\partial^{\text{2}}f_{F}\left(z,t\right)}{\partial t^{\text{2}}}=F\left(z,t\right)$$ (5)

where $f_{F}\left(z,t\right)$ represents the vibration output of the beam, ρ is the material density of the beam, and F(z,t) is the external load. Based on Eq (3), the following relations are derived:


$$A\left(z\right)=\beta z^{4/3}$$ (6)

$$I_{s}\left(z\right)=\frac{A(z{)}^{\text{2}}}{4\pi }=\frac{\beta^{\text{2}}z^{8/3}}{4\pi }$$ (7)

where A(z) is the cross-sectional area of the fruit branch, and $I_{s}\left(z\right)$ is its moment of inertia. The parameter $\beta =\frac{\pi }{\text{4}}{\left(\frac{16q}{\pi \sigma_{\text{max}}}\right)}^{\text{2}/3}$ is a sectional proportionality coefficient, which can be obtained by solving the following equations: $A_{\text{1}}=\beta z_{\text{1}}^{4/3}$, $A_{\text{2}}=\beta z_{\text{2}}^{4/3}$, $z_{\text{2}}-z_{\text{1}}=L$. Here $A_{\text{1}}$ and $A_{\text{2}}$ are the cross-sectional areas at the top and root of branch respectively, $z_{\text{1}}$ and $z_{\text{2}}$ are their corresponding coordinates, L=0.885m represents the length of the fruit branch, $d_{\text{1}}=9.3\text{mm}$ is the basal diameter, $d_{\text{2}}=2.9\text{mm}$ is the apical diameter. the value for these geometric parameters can be obtained from Table 1. Substituting the values yields: $A_{\text{1}}=\pi \cdot 0.00145^{\text{2}}\approx 6.605\times 10^{-6}{\text{m}}^{\text{2}}$, $A_{\text{2}}=\pi \cdot 0.00465^{\text{2}}\approx 6.789\times 10^{-5}{\text{m}}^{\text{2}}$. Solving this equation gives $\beta \approx 6.18\times 10^{-5}{\text{m}}^{2/3}$.

When the plant is subjected to the force Fsinωt from the harvesting finger, the branch exhibits a vibration output response. Thus, Eq (5) becomes:


$$\frac{\partial^{\text{2}}}{\partial z^{\text{2}}}\left[\frac{E\beta^{\text{2}}z^{8/3}}{4\pi }\frac{\partial^{\text{2}}f_{F}\left(z,t\right)}{\partial z^{\text{2}}}\right]+\rho \beta z^{4/3}\frac{\partial^{\text{2}}f_{F}\left(z,t\right)}{\partial t^{\text{2}}}=Fsin\omega t$$ (8)

where ω is the frequency of the excitation force from the harvesting finger striking the branch, and F is the amplitude of the excitation force. Due to the presence of damping in the branch vibration, the transient response of the forced vibration fades away over time. To simplify the analysis, this study focuses on the steady-state response. Assuming the vibration frequency of the branch matches the excitation frequency of the harvesting finger, the steady-state response output of the branch is . Defining the fixed end of the branch at $z_{\text{1}}$, the solution to Eq (8) is assumed to take the form of a power series:


$$g\left(z\right)=\sum_{n=0}^{\infty }a_{n}{\left(z-z_{\text{1}}\right)}^{\frac{4n}{\text{3}}}$$ (9)

Substituting Eq (9) into Eq (8) and resolving the relationships between the series coefficients, the steady-state response output is determined as:


$$f\left(z,t\right)=sin\omega t\sum_{k=0}^{\infty }b_{k}{\left(z-z_{\text{1}}\right)}^{\frac{8k+4}{\text{3}}}$$ (10)

where $b_{\text{0}}=\frac{9\pi F}{2E\beta^{\text{2}}}$, and $b_{k}=b_{\text{0}}G^{k}\prod_{j=1}^{k}c_{2j},k\geq 1$, $c_{2j}=\frac{81}{4(2j+1)(8j+1)(8j+6)(8j+3)}$. Eq (10) represents the solution for the stable vibration of the fruit branch under the action of the harvesting finger. Expanding the terms of Eq (10) yields:


$$f\left(z,t\right)=sin\omega t\left[\frac{9\pi F}{2E\beta^{\text{2}}}(z-z_{\text{1}}{)}^{4/3}+\frac{27\pi^{\text{2}}F\rho \omega^{\text{2}}}{308E^{\text{2}}\beta^{\text{3}}}(z-z_{\text{1}}{)}^{\text{4}}+\cdots \right]$$ (11)

when ω is small, the second term in Eq (11) can be neglected. The analysis shows that at low excitation frequency, the vibration of the fruit branch can be approximated as a sinusoidal motion, standardized as , where $A_{s}=\frac{9\pi F}{2E\beta^{\text{2}}}(z-z_{\text{1}}{)}^{4/3}$ is the vibration amplitude.

#### 2.2.3. Dynamic model of the fruit stem.

The four fundamental modes of general fruit motion include flapping, pendulum, rotation, and torsion. These modes are widely used to simplify the kinematic analysis of fruit vibration [17]. Crooke and Rand [4] developed a three-degree-of-freedom model that identified the characteristics of excitation frequencies that lead to system instability. However, their study did not account for the influence of the initial angles of the stem and branch on fruit detachment. Furthermore, since the frequency of the out-of-phase mode is significantly higher than that of the in-phase mode, and considering the fragile nature of Blue Honeysuckle, the harvesting excitation frequency would not approach the out-of-phase mode frequency. Therefore, it is necessary to investigate the precise detachment mechanism of the fruit when the stem and branch have different initial angles.

**Fig 3:**
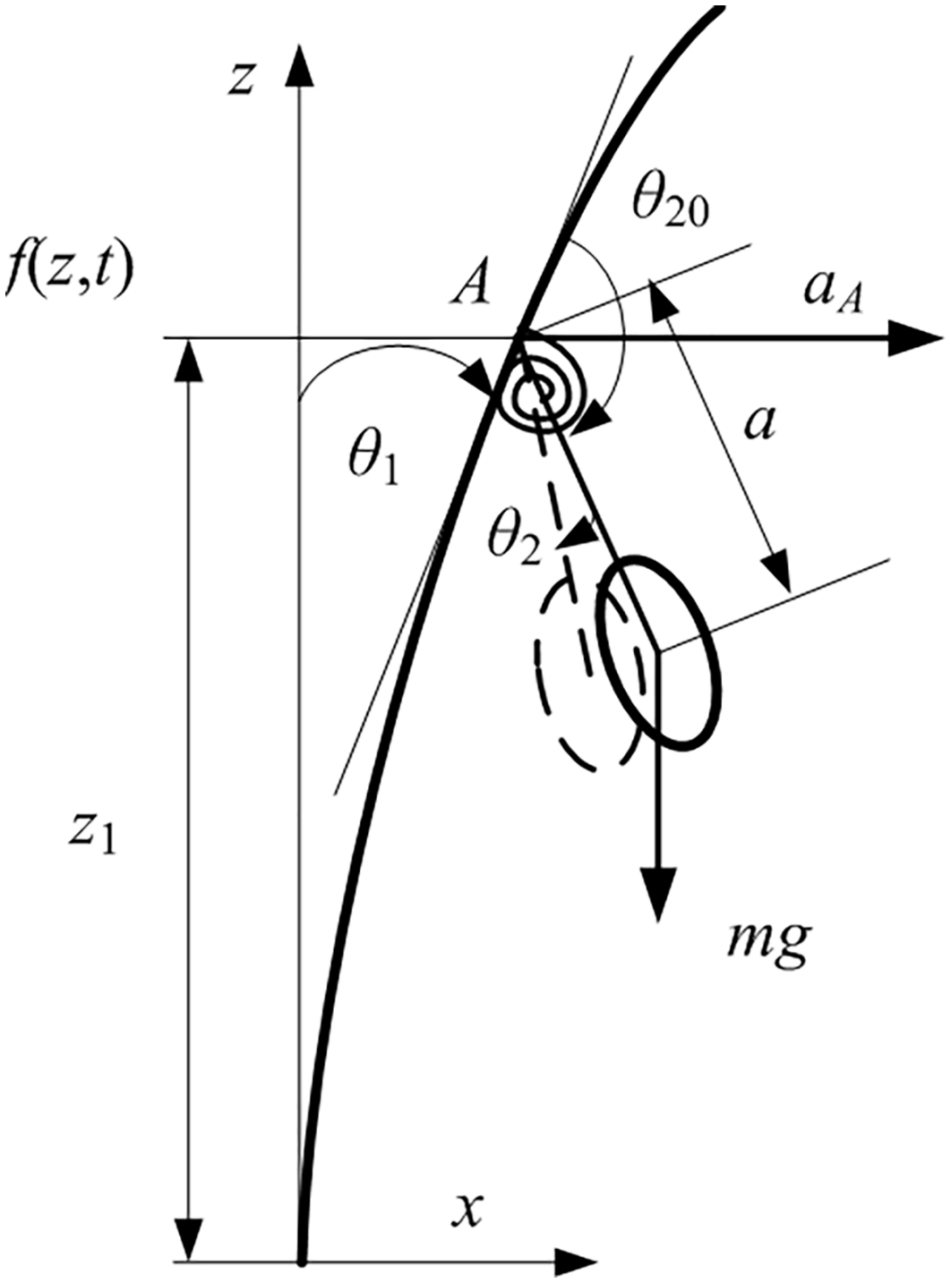
A schematic diagram of the vibration of the fruit-stem.

The stem is approximated as a cantilever beam connected to the branch. The bending moment at the junction between the fruit and the stem is less than that at the connection between the stem and the branch. During vibratory harvesting, the rotation angle at the fruit-stem junction is relatively small. Thus, the stem and the fruit can be treated as a rigid body connected to the branch via a torsional spring. This means that among the four fundamental fruit motion modes, only the pendulum mode is considered. Additionally, note that there is an initial angle $\theta_{20}$ between the stem and the branch. A schematic diagram of the simplified structure is shown in Fig 3.

Under the assumptions that the fruit-stem and the branch lie in the same plane and that the branch undergoes small amplitude vibrations, the acceleration $a_{A}$ of point A on the branch is aligned with the x -axis. When the mass of the fruit stem is neglected, the angular momentum equation of the fruit-stem about the moving point A is given by:


$$\left(I+ma^{\text{2}}\right)\left(\frac{{\text{d}}^{\text{2}}\theta_{\text{2}}}{\text{d}t^{\text{2}}}+\frac{{\text{d}}^{\text{2}}}{\text{d}t^{\text{2}}}\frac{\partial f}{\partial z}\right)-ma\frac{{\text{d}}^{\text{2}}f}{\text{d}t^{\text{2}}}cos\left(\theta_{\text{2}}+\theta_{20}+\frac{\partial f}{\partial z}\right)=mgasin\left(\theta_{\text{2}}+\theta_{20}+\frac{\partial f}{\partial z}\right)-K\theta_{\text{2}}-\mu \frac{\text{d}\theta_{\text{2}}}{\text{d}t}$$ (12)

where $\frac{\partial f}{\partial z}=\theta_{\text{1}}$ is the rotation angle of the branch, I is the mass moment of inertia of the fruit about its center of mass, a is the distance of the fruit’s center of mass from point A, and $a_{A}$ is the acceleration of the moving point A with magnitude $\frac{{\text{d}}^{\text{2}}f}{\text{d}t^{\text{2}}}$, which defines the excitation direction. $K\theta_{\text{2}}+\mu \frac{\text{d}\theta_{\text{2}}}{\text{d}t}$ represents the moment due to elastic and damping forces of the branch about point A. $\theta_{\text{2}}$ denotes the angle deviation of the branch from its equilibrium position. The angle between stem and excitation direction is $\theta_{\text{1}}\text{+}\theta_{\text{2}}+\theta_{20}-\frac{\pi }{\text{2}}$. When the excitation frequency is low, the branch vibration f exhibits a sinusoidal behavior.

In Eq (12), K is the spring stiffness coefficient of the fruit stem. Based on the beam bending deformation equation $EI_{b}\frac{\text{d}\theta_{\text{2}}}{\text{d}l}=M$, it can be approximated that $K=\frac{EI_{b}}{l}$, where l is the length of the fruit stem, $I_{b}$ is the area moment of inertia of the fruit stem cross-section about the centroidal axis, and M is the bending moment of the fruit stem cross-section. Considering that $\frac{\partial f}{\partial z}$ is small and its second-order time derivative can be neglected. Eq (12) can be approximated as:


$$J\frac{d^{\text{2}}\theta_{\text{2}}}{dt^{\text{2}}}+ma\omega^{\text{2}}fcos\left(\theta_{\text{2}}+\theta_{20}\right)=mgasin\left(\theta_{\text{2}}+\theta_{20}\right)-K\theta_{\text{2}}-\mu \frac{\text{d}\theta_{\text{2}}}{\text{d}t}$$ (13)

where $J=I+ma^{\text{2}}$. Eq (13) is a nonlinear equation, and approximate solutions can be obtained using different simplification methods.

**2.2.3.1. Vibration amplitude of stem when the stem is perpendicular to excitation direction.** When the stem is perpendicular to excitation direction and perpendicular to the ground, here $\theta_{20}\text{=}\pi$, Eq (13) is written as:


$$J\frac{d^{\text{2}}\theta_{\text{2}}}{dt^{\text{2}}}\text{+}\mu \frac{\text{d}\theta_{\text{2}}}{\text{d}t}\text{+}K\theta_{\text{2}}+mgasin\theta_{\text{2}}=ma\omega^{\text{2}}fcos\theta_{\text{2}}$$ (14)

Assuming small-angle oscillations of the fruit stem, substituting $cos\theta_{\text{2}}\approx 1$ and $sin\theta_{\text{2}}\approx 0$ into Eq (14) yields:


$$J\frac{d^{\text{2}}\theta_{\text{2}}}{dt^{\text{2}}}+\mu \frac{\text{d}\theta_{\text{2}}}{\text{d}t}+K\theta_{\text{2}}=ma\omega^{\text{2}}f$$ (15)

Substituting into Eq (15) gives:


$$J\frac{d^{\text{2}}\theta_{\text{2}}}{dt^{\text{2}}}+\mu \frac{\text{d}\theta_{\text{2}}}{\text{d}t}+K\theta_{\text{2}}=ma\omega^{\text{2}}A_{s}sin\omega t$$ (16)

Eq (16) is a linear nonhomogeneous equation. Its steady-state solution corresponds to a particular integral of the form:


$$\theta_{\text{2}}\left(t\right)=A_{\text{1}}sin\omega t+A_{\text{2}}cos\omega t$$ (17)

where $A_{\text{1}}$ and $A_{\text{2}}$ are constants to be determined. Substituting Eq (17) into Eq (16) and equating the coefficients of the sinωt and cosωt yields the steady-state solution:


$$\theta_{\text{2}}\left(t\right)=\frac{ma\omega^{\text{2}}A_{s}}{\sqrt{(K-J\omega^{\text{2}}{)}^{\text{2}}+(\mu \omega {)}^{\text{2}}}}sin\left[\omega t-arctan\left(\frac{\mu \omega }{K-J\omega^{\text{2}}}\right)\right]$$ (18)

Eq (18) describes the steady-state vibrational response of the fruit-stem system. A larger vibration amplitude $A_{s}$ of the fruit branch leads to an increased amplitude of angular oscillation of the stem.

**2.2.3.2. Vibration amplitude of stem when the stem is parallel to excitation direction.** When the stem is parallel to the excitation direction and is horizontal to the ground, $\theta_{20}\text{=}\frac{\pi }{\text{2}}$, Eq (13) is written as:


$$J\frac{d^{\text{2}}\theta_{\text{2}}}{dt^{\text{2}}}+\mu \frac{\text{d}\theta_{\text{2}}}{\text{d}t}+K\theta_{\text{2}}=ma\omega^{\text{2}}fsin\theta_{\text{2}}+mgacos\theta_{\text{2}}$$ (19)

Assuming small angle vibrations, where $cos\theta_{\text{2}}\approx 1$, $sin\theta_{\text{2}}\approx \theta_{\text{2}}$, Eq (19) is written as:


$$J\frac{d^{\text{2}}\theta_{\text{2}}}{dt^{\text{2}}}+\mu \frac{\text{d}\theta_{\text{2}}}{\text{d}t}+K\theta_{\text{2}}-ma\omega^{\text{2}}A_{s}sin\omega t\theta_{\text{2}}=mga$$ (20)

Eq (20) represents a linear non-homogeneous differential equation that contains a time-varying coefficient term $-ma\omega^{\text{2}}A_{s}sin\omega t\theta_{\text{2}}$, indicating the system is subjected to parametric excitation. Due to this parametric excitation term, the steady-state response includes components at the fundamental frequency ω and may also include a constant term and higher harmonics. To simplify the analysis, it is assumed that the steady-state solution comprises a constant term and the fundamental frequency component, while higher harmonics are neglected. Therefore, the steady-state solution is assumed to take the form:


$$\theta_{\text{2}}\left(t\right)=\theta_{\text{0}}+\theta_{c}cos\omega t+\theta_{s}sin\omega t$$ (21)

where $\theta_{\text{0}}$ is the constant offset, and $\theta_{c}$, $\theta_{s}$ are amplitude coefficients. Substituting Eq (21) into Eq (20) and equating the coefficients of the same frequency terms, the following is obtained:


$$\theta_{s}=\frac{ma\omega^{\text{2}}A_{s}\left(K-J\omega^{\text{2}}\right)}{D}\theta_{\text{0}}$$ (22)

where $D={\left(K-J\omega^{\text{2}}\right)}^{\text{2}}+\mu^{\text{2}}\omega^{\text{2}}$. Furthermore:


$$\theta_{c}=-\frac{\mu \omega^{\text{3}}maA_{s}}{D}\theta_{\text{0}}$$ (23)

$$\theta_{\text{0}}=\frac{2mgaD}{2DK-(ma\omega^{\text{2}}A_{s}{)}^{\text{2}}\left(K-J\omega^{\text{2}}\right)}$$ (24)

When the excitation frequency ω→∞, the constant offset $\theta_{\text{0}}\to \text{0}$. Thus, the steady-state solution is:


$$\theta_{\text{2}}\left(t\right)=\theta_{\text{0}}+\frac{ma\omega^{\text{2}}A_{s}\theta_{\text{0}}}{\sqrt{{\left(K-J\omega^{\text{2}}\right)}^{\text{2}}+\mu^{\text{2}}\omega^{\text{2}}}}sin\left[\omega t-arctan\left(\frac{\mu \omega }{K-J\omega^{\text{2}}}\right)\right]$$ (25)

The steady-state solution contains a constant term and an oscillatory term at frequency ω. If the constant offset becomes unbounded, $\theta_{\text{0}}\to \infty$, it indicates that the system is unstable, and the stem fractures due to excessive vibration amplitude. The condition for fruit-stem system instability is:


$$K-\frac{\text{1}}{\text{2}}\frac{{\left(ma\omega^{\text{2}}A_{s}\right)}^{\text{2}}\left(K-J\omega^{\text{2}}\right)}{{\left(K-J\omega^{\text{2}}\right)}^{\text{2}}+\mu^{\text{2}}\omega^{\text{2}}}=0$$ (26)

Let the damping ratio be $\zeta =\frac{\mu }{\sqrt{\text{2}KJ}}$. The relationship between the damping ratio and the frequency at the onset of instability is:


$$\zeta^{\text{2}}\text{=}\frac{{\left(ma\omega^{\text{2}}A_{s}\right)}^{\text{2}}\left(K-J\omega^{\text{2}}\right)-\text{2}K{\left(K-J\omega^{\text{2}}\right)}^{\text{2}}}{8JK^{\text{2}}\omega^{\text{2}}}$$ (27)

When the damping ratio is zero, Eq (27) becomes:


$${\left(ma\omega^{\text{2}}A_{s}\right)}^{\text{2}}\left(K-J\omega^{\text{2}}\right)=2K{\left(K-J\omega^{\text{2}}\right)}^{\text{2}}$$ (28)

If $K-J\omega^{\text{2}}\neq 0$, simplifying (28) yields:


$$\omega^{\text{2}}=\omega_{\text{0}}^{\text{2}}\chi \left(\sqrt{\chi^{\text{2}}+2}-\chi \right)$$ (29)

where $\chi =\frac{J}{maA_{s}}$. When the system satisfies this frequency condition, it becomes unstable, and the fruit is detached due to the unstable vibration of the stem.

**2.2.3.3 Vibration amplitude of stem when the stem has a small angle to the excitation direction.** When the angle $\theta_{20}$ is close to $\frac{\pi }{\text{2}}\text{+}\delta$ and the fruit stem has a small angle δ relative to the horizontal, Eq (13) simplifies to:


$$J\frac{d^{\text{2}}\theta_{\text{2}}}{dt^{\text{2}}}+\mu \frac{\text{d}\theta_{\text{2}}}{\text{d}t}+K\theta_{\text{2}}=ma\omega^{\text{2}}fsin\left(\theta_{\text{2}}\text{+}\delta \right)+mgacos\left(\theta_{\text{2}}\text{+}\delta \right)$$ (30)

For small-angle vibrations, where and , Eq (30) is written as:


$$J\frac{d^{\text{2}}\theta_{\text{2}}}{dt^{\text{2}}}+\mu \frac{\text{d}\theta_{\text{2}}}{\text{d}t}+K\theta_{\text{2}}-ma\omega^{\text{2}}A_{s}sin\omega t\theta_{\text{2}}=mga\text{+}ma\omega^{\text{2}}A_{s}sin\omega t\delta$$ (31)

Eq (31) represents a linear nonhomogeneous differential equation that includes a parametric excitation term ($-ma\omega^{\text{2}}A_{s}sin\omega t\theta_{\text{2}}$). Assuming the steady-state solution comprises a constant term and the fundamental frequency component while ignoring higher harmonics, the solution is assumed to take the form:


$$\theta_{\text{2}}\left(t\right)=\delta_{\text{0}}+\delta_{s}sin\omega t+\delta_{c}cos\omega t$$ (32)

where, $\delta_{\text{0}}$ is the constant offset, $\delta_{c}$ and $\delta_{s}$ are amplitude coefficients, all of which are undetermined constants. Substituting Eq (32) into Eq (31) and equating coefficients for terms of the same frequency, the constant offset is obtained as:


$$\delta_{\text{0}}=\frac{2mgaD+{\left(ma\omega^{\text{2}}A_{s}\right)}^{\text{2}}\left(K-J\omega^{\text{2}}\right)\delta }{2DK-{\left(ma\omega^{\text{2}}A_{s}\right)}^{\text{2}}\left(K-J\omega^{\text{2}}\right)}$$ (33)

The undetermined coefficients are:


$$\delta_{s}=\frac{2ma\omega^{\text{2}}A_{s}\left(K\delta +mag\right)\left(K-J\omega^{\text{2}}\right)}{2DK-{\left(ma\omega^{\text{2}}A_{s}\right)}^{\text{2}}\left(K-J\omega^{\text{2}}\right)}$$ (34)

$$\delta_{c}=-\frac{\mu \omega }{K-J\omega^{\text{2}}}\delta_{s}$$ (35)

Thus, the vibration solution takes the form:


$$\theta_{\text{2}}\left(t\right)=\delta_{\text{0}}+\frac{2ma\omega^{\text{2}}A_{s}\left(K\delta +mag\right)\sqrt{{\left(K-J\omega^{\text{2}}\right)}^{\text{2}}+\mu^{\text{2}}\omega^{\text{2}}}}{2DK-{\left(ma\omega^{\text{2}}A_{s}\right)}^{\text{2}}\left(K-J\omega^{\text{2}}\right)}sin\left[\omega t-arctan\left(\frac{\mu \omega }{K-J\omega^{\text{2}}}\right)\right]$$ (36)

The fruit stem vibrates about a constant offset angle. The magnitude of this offset is positively correlated with the initial angle; a larger initial angle results in a larger offset. Furthermore, when the excitation frequency is generally below the natural frequency of the fruit stem ($K-J\omega^{\text{2}}>0$), the constant offset of the fruit stem is greater than when δ=0. According to Eq (33), if $\delta_{\text{0}}\to \infty$, it indicates system instability. The instability threshold for the fruit stem vibration, $2DK-(ma\omega^{\text{2}}A_{s}{)}^{\text{2}}\left(K-J\omega^{\text{2}}\right)$, remains unchanged from the case when δ=0, meaning the instability of the fruit stem is independent of δ. If $K-J\omega^{\text{2}}>0$, both the constant offset and the angular amplitude of the fruit stem are greater than the values obtained when δ=0. This implies that fruit detachment through vibrational excitation becomes more difficult when the stem is parallel to the excitation direction.

#### 2.2.4. Force analysis of the stem.

**Fig 4:**
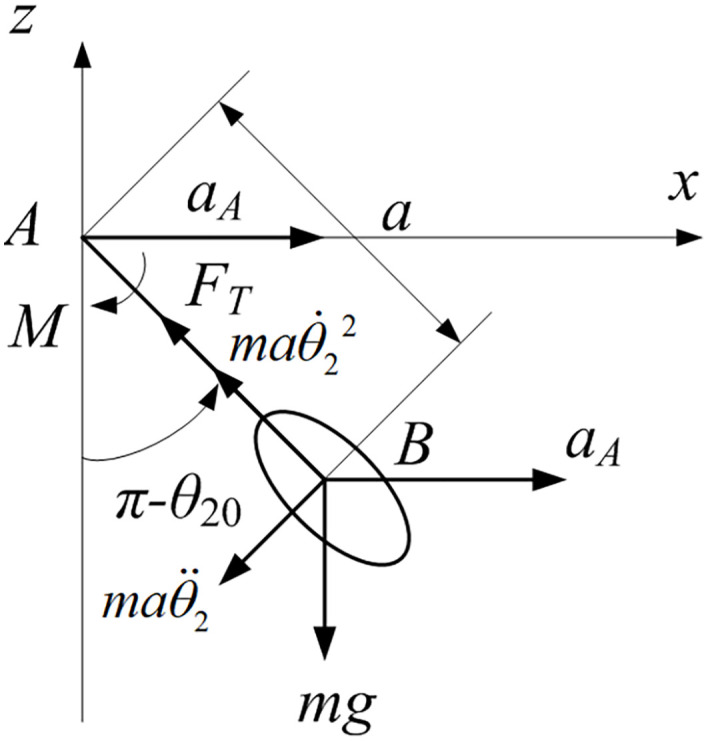
Force analysis diagram of the fruit stem.

When the fruit-stem system oscillates together with the fruit branch under a small rotation angle, the forces acting on the stem are illustrated in Fig 4. Only the axial tension and internal resisting moment of the stem are considered.

The axial force $F_{T}$ along the fruit stem is determined from the acceleration at point B as:


$$F_{T}-mgcos\left(\pi -\theta_{2\text{0}}\right)-ma_{A}sin\left(\pi -\theta_{2\text{0}}\right)=ma{{\dot{\theta }}_{\text{2}}}^{\text{2}}$$ (37)

The moment at point A within the fruit stem is given by:


$$M=K\theta_{\text{2}}\text{+}\mu \frac{\text{d}\theta_{\text{2}}}{\text{d}t}+mgasin\left(\pi -\theta_{2\text{0}}\right)$$ (38)

Fruit detachment occurs when the maximum stress within the fruit stem exceeds its ultimate stress. This condition satisfies:


$$\sigma_{max}\geq \left[\sigma \right]$$ (39)

Since the fruit stem is subjected to both axial force and bending moment, the combined stress is expressed as:


$$\sigma \text{=}\frac{F_{T}}{S}+\frac{M}{W}$$ (40)

**2.2.4.1 Axial force in the fruit stem.** The maximum axial force $F_{Tmax}$ along the axial direction of the fruit stem is determined based on its angular amplitude $\theta_{\text{2}}\left(t\right)$. When the stem is perpendicular to the excitation direction, the angular velocity is obtained from Eq (18) as:


$${\dot{\theta }}_{\text{2}}\left(t\right)=\frac{ma\omega^{\text{3}}A_{s}}{\sqrt{(K-J\omega^{\text{2}}{)}^{\text{2}}+(\mu \omega {)}^{\text{2}}}}cos\left(\omega t-arctan\left(\frac{\mu \omega }{K-J\omega^{\text{2}}}\right)\right)$$ (41)

At the moment of fruit detachment due to vibration, where $\theta_{20}\text{=}\pi$, , Eq (37) yields the maximum axial force exerted on the fruit stem as:


$$F_{Tmax}\text{=}mg+ma{\dot{\theta }}_{2max}^{\text{2}}\text{=}mg+ma{\left(\frac{ma\omega^{\text{3}}A_{s}}{\sqrt{(K-J\omega^{\text{2}}{)}^{\text{2}}+(\mu \omega {)}^{\text{2}}}}\right)}^{\text{2}}$$ (42)

When the stem is parallel to the excitation direction, if the value of $\theta_{\text{0}}$ is finite, Eq (25) gives:


$${\dot{\theta }}_{2max}=\frac{ma\omega^{\text{3}}A_{s}\theta_{\text{0}}}{\sqrt{(K-J\omega^{\text{2}}{)}^{\text{2}}+(\mu \omega {)}^{\text{2}}}}$$ (43)

Similarly, where $\theta_{20}\text{=}\frac{\pi }{\text{2}}$, the maximum axial force experienced by the fruit stem is:


$$F_{Tmax}\text{=}ma_{A}+ma{\dot{\theta }}_{2max}^{\text{2}}=m\omega^{\text{2}}A_{s}+ma{\left(\frac{ma\omega^{\text{3}}A_{s}\theta_{\text{0}}}{\sqrt{(K-J\omega^{\text{2}}{)}^{\text{2}}+(\mu \omega {)}^{\text{2}}}}\right)}^{\text{2}}$$ (44)

Eqs.(42) and (44)represent the forces at the position where the angular velocity is maximum.

If the stem has a small angle δ relative to the excitation direction, both the constant offset and the angular amplitude of the fruit stem, according to Eq (36), are greater than those when the stem is parallel to the excitation direction. For cases where the stem is positioned between perpendicular and parallel to the excitation direction, if the $ma_{A}$ and mg terms are neglected, the maximum axial force in the fruit stem under the same excitation frequency lies between those given by Eqs.(42) and(44).

**2.2.4.2. Bending moment in the fruit stem.** If the fruit stem bends, the maximum bending moment it experiences is determined. When the stem is perpendicular to the excitation direction, the bending moment is obtained from Eq (38) as described by:


$$M_{A}=K\theta_{\text{2}}\text{+}\mu {\dot{\theta }}_{\text{2}}$$ (45)

The maximum bending moment in the fruit stem is:


$$M_{Amax}=\frac{ma\omega^{\text{2}}A_{s}\sqrt{K^{\text{2}}+(\mu \omega {)}^{\text{2}}}}{\sqrt{(K-J\omega^{\text{2}}{)}^{\text{2}}+(\mu \omega {)}^{\text{2}}}}$$ (46)

When the stem is parallel to the excitation direction and the value of $\theta_{\text{0}}$ is finite, the bending moment in the fruit stem is:


$$M_{A}=K\theta_{\text{2}}\text{+}\mu {\dot{\theta }}_{\text{2}}+mga$$ 47)

Because the offset of the fruit stem is small and the gravitational moment is neglected, the maximum bending moment in the fruit stem is:


$$M_{Amax}=\frac{ma\omega^{\text{2}}A_{s}\theta_{\text{0}}\sqrt{K^{\text{2}}+(\mu \omega {)}^{\text{2}}}}{\sqrt{(K-J\omega^{\text{2}}{)}^{\text{2}}+(\mu \omega {)}^{\text{2}}}}$$ (48)

If the stem has a small angle δ with the excitation direction, according to Eq (36), the value of the maximum bending moment lies between those given by Eqs.(46) and (48). If the damping is small, the expressions in Eqs.(46) and (48)reach their maxima where the angular amplitude $\theta_{\text{2}}\left(t\right)$ is maximized.

For simplicity of analysis, only the case of maximum angular velocity is considered, yields:


$$\sigma_{max}\text{=}\frac{F_{Tmax}}{S}$$ (49)

Similarly, when the maximum angular amplitude occurs, the relationship is:


$$\sigma_{max}\text{=}\frac{M_{max}}{W}$$ (50)

Regarding fruit detachment, it suffices to consider the maximum tensile force and the maximum bending moment in the fruit stem.

#### 2.2.5. Vibration of fruit branch and stem based on numerical analysis.

Using the data in Table 1, the section proportionality factor of the branch β is calculated. This calculated result is then substituted into Eq (11) to obtain the simulated forced steady-state vibration response curves of the fruit branch. Using the same dataset, the amplitude of the fruit stem during its steady-state vibration response is calculated as a function of the harvesting vibration excitation frequency by substituting the values into Eqs (18) and (25).

Furthermore, these data are used in conjunction with Eqs (42),(44),(46), and (48) to plot the dependence of the excitation frequency on both the maximum axial force and the maximum bending moment in the fruit stem during its steady-state response. All the simulations are performed using Mathematica.

A finite element model incorporating the physical properties of the fruit stem and fruit (from Table 1) is developed. The model is then imported into ABAQUS for finite element analysis to determine the amplitude of the fruit stem and the resulting von Mises stress as functions of different excitation frequencies and at different angles of the stem relative to the excitation direction.

## 3. Results

### 3.1. Vibration amplitude characteristics of fruit branches

**Fig 5:**
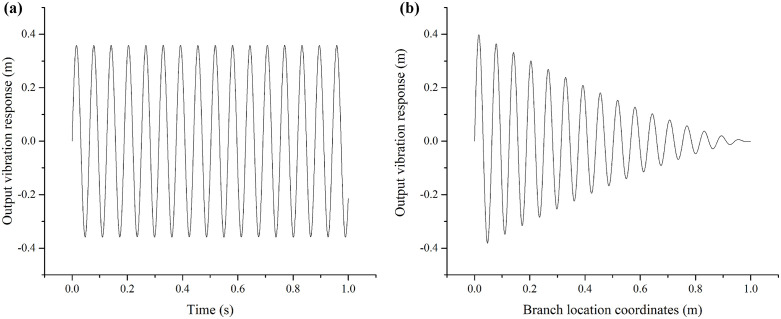
Simulation time-domain dynamic characteristics of steady-state vibration for the fruit branch. (a) Simulation curve of branch-tip vibration. (b) Vibration response at different branch positions.

The simulation results of the forced steady-state vibration of the Blue Honeysuckle fruit branch are presented in Fig 5. Fig 5(a) shows that the vibration frequency of the fruit branch matches the excitation frequency, indicating that the branch undergoes simple harmonic motion. Furthermore, Fig 5(b) demonstrates that the vibration amplitude increases progressively from the fixed end to the free end of the branch.

### 3.2. Vibration amplitude of fruit stem

#### 3.2.1. Amplitude of fruit stem when its longitudinal axis perpendicular to excitation direction.

**Fig 6:**
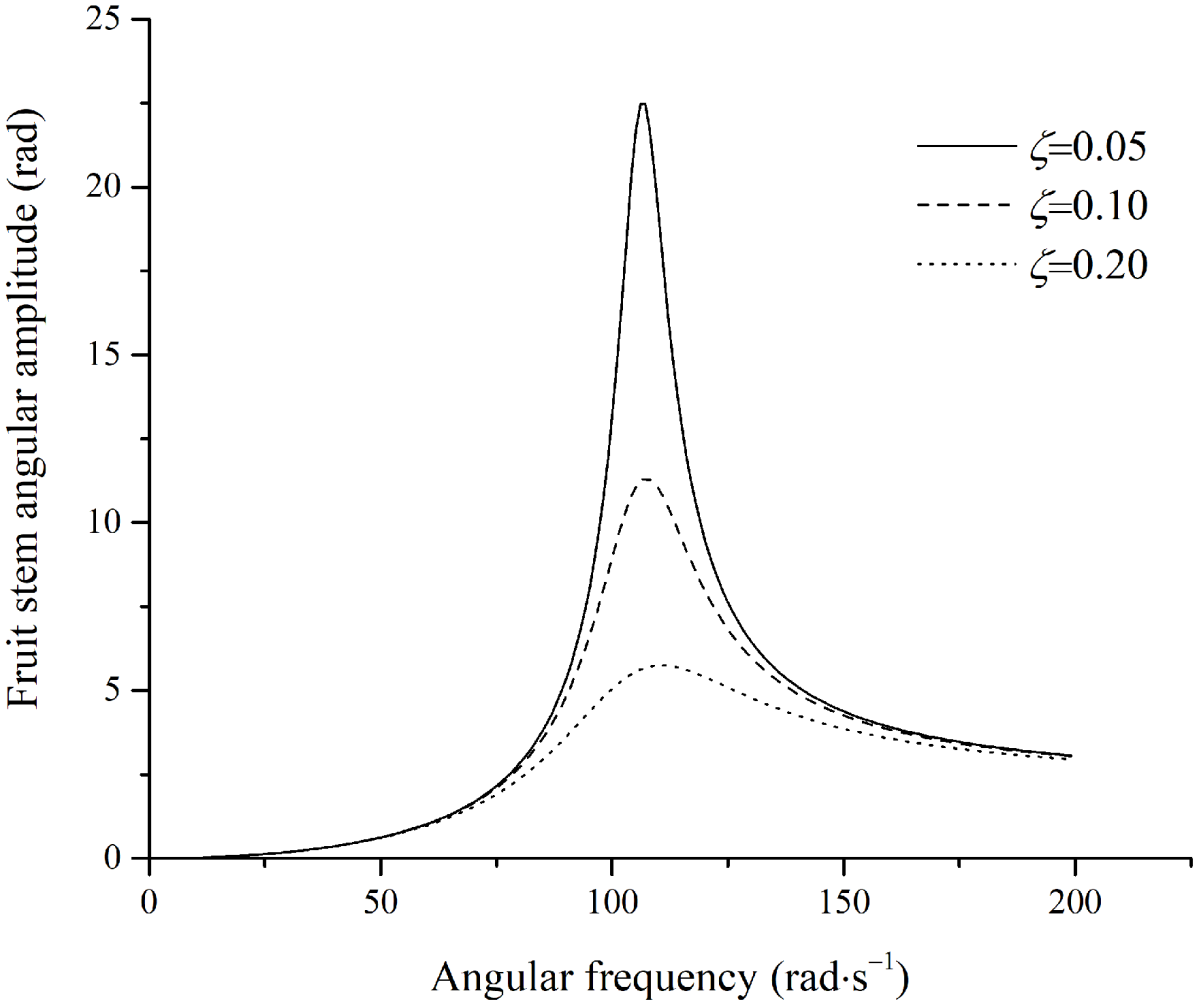
Angular amplitude of stem perpendicular to the excitation direction.

Based on the data from Table 1 and Eq (18), the amplitude of the fruit branch was set to 39mm for subsequent calculations. Using the excitation frequency of the fruit stem as the independent variable, simulation curves for the angular amplitude of the fruit stem under steady-state conditions were generated, as shown in Fig 6.

The relationship between the damping ratio and the damping coefficient is given by $\zeta \text{=}\frac{\mu }{\sqrt{\text{2}KJ}}$. In Fig 6, at a given excitation frequency, a lower damping ratio results in a larger vibration amplitude of the fruit stem. When the damping coefficient of the fruit stem is relatively small, the amplitude initially increases and then decreases with increasing excitation frequency, which is characteristic of a system approaching its natural frequency. The maximum amplitude occurs when the excitation frequency approaches the natural frequency of the fruit-stem system. At very high excitation frequencies, however, the amplitude asymptotically approaches a specific value, $\frac{maA_{s}}{J}$, which is independent of the damping ratio. This asymptotic value is determined solely by the system inertia and the excitation parameters, specifically J and $maA_{s}$, rather than by the excitation frequency or damping coefficient. This phenomenon occurs because, under high-frequency excitation, the system’s inertial response is dominated by the term $J\omega^{\text{2}}$, which counterbalances the excitation force component $ma\omega^{\text{2}}A_{s}$, leading to an amplitude approaching a constant value.

#### 3.2.2. Amplitude of the fruit stem when the stem is parallel to excitation direction.

**Fig 7:**
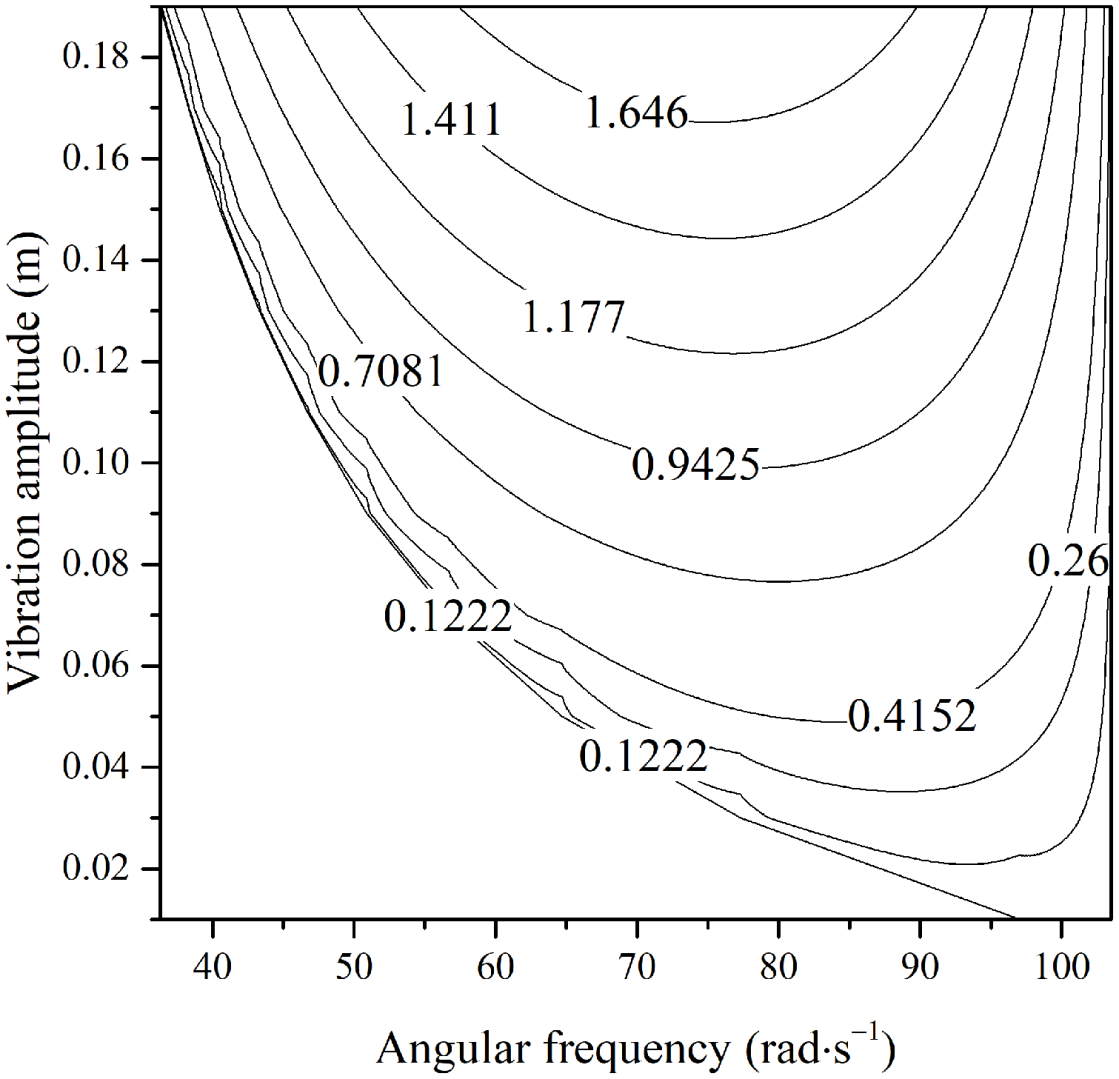
Unstable region of the fruit stem vibration under different stem damping ratio.

The coupling between constant excitation mga and parametric excitation terms $-ma\omega^{\text{2}}A_{s}sin\omega t\theta_{\text{2}}$ may induce vibrational instability in the fruit stem. The unstable region of the fruit-stem system, determined using Eq (27), is shown in Fig 7, from which the unstable region of vibration can be identified.

**Fig 8:**
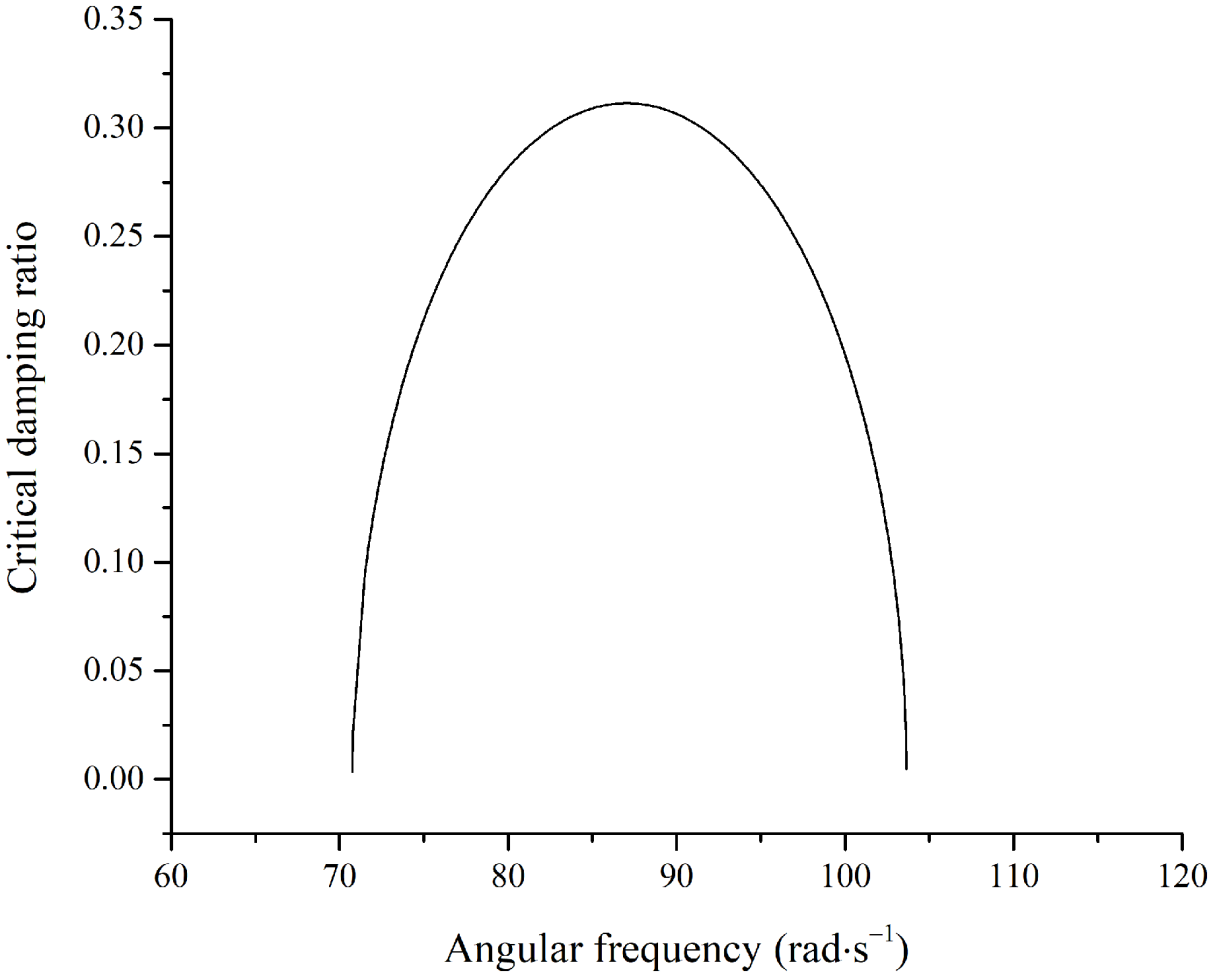
Relationship between the excitation frequency and damping ratio.

Fig 7 shows that a larger excitation amplitude results in a lower critical unstable angular frequency of the fruit stem at a given damping ratio, which is lower than the natural frequency of the fruit-stem system. For a damping ratio of zero, the relationship between the excitation frequency and amplitude at the onset of instability is given by Eq (29). With the fruit branch amplitude set to 39mm, the relationship between the excitation frequency and damping ratio at the instability threshold is plotted in Fig 8.

**Fig 9:**
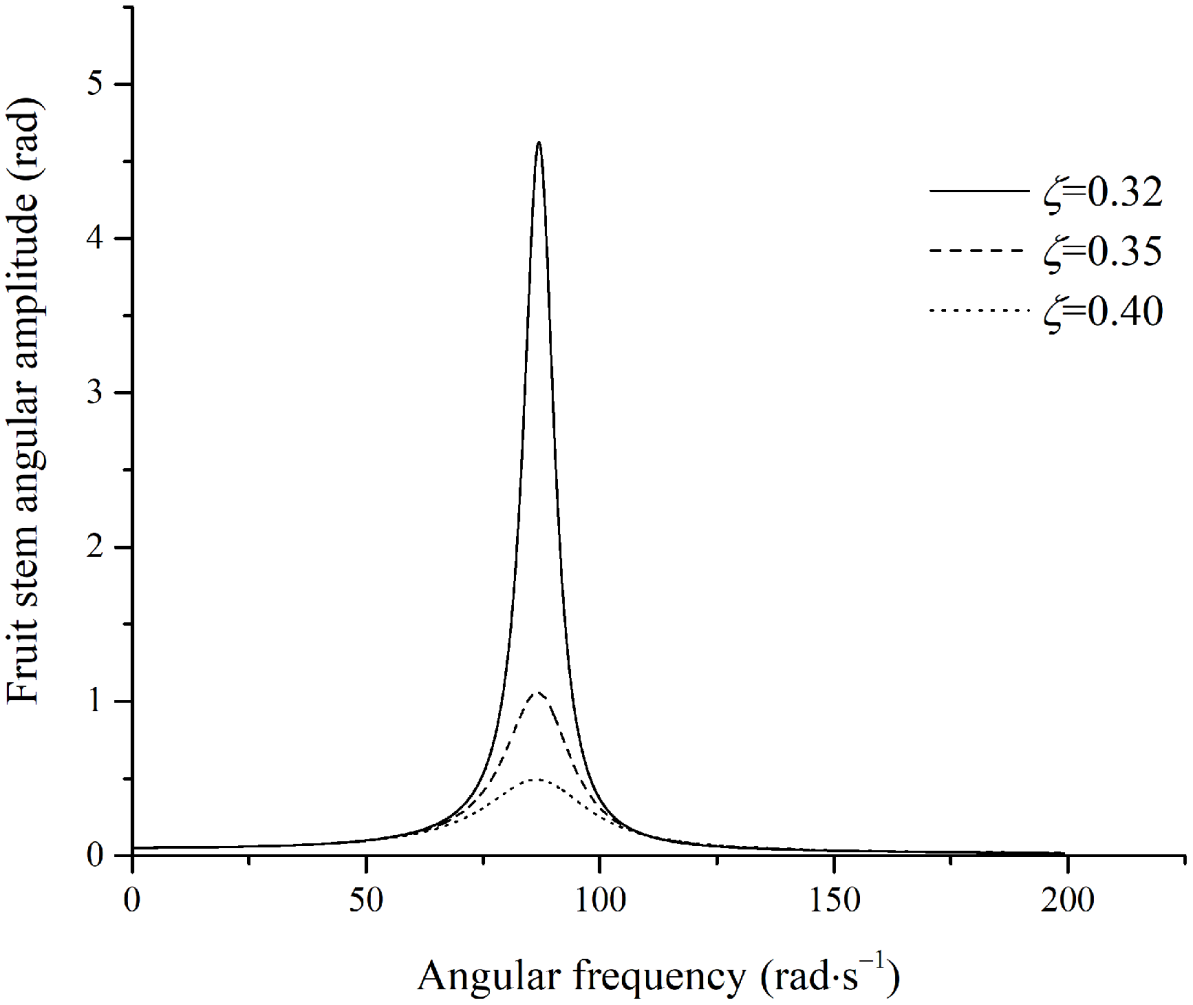
Angular amplitude of stem parallel to the excitation direction.

The unstable region for fruit stem vibration lies below the curve in Fig 8. When the damping ratio of the fruit stem is less than approximately 0.32, an unstable region exists. At a damping ratio of zero, the unstable region spans from $\text{70.9rad}\cdot {\text{s}}^{-\text{1}}$ to $\text{103.6rad}\cdot {\text{s}}^{-\text{1}}$. Based on the parameters in Table 1, the calculated natural frequency is $\omega_{\text{0}}\text{=103.6rad/s}$. In this case, the unstable frequency range is lower than the natural frequency, i.e., $\omega \leq \omega_{\text{0}}$. To examine the stable motion characteristics of the fruit stem, damping ratios of 0.32, 0.35, and 0.40 were selected to plot the relationship between the vibration amplitude of the fruit stem and the excitation frequency, as shown in Fig 9.

Owing to variations in damping ratio, the angular amplitude of the fruit-stem system varies significantly within the excitation frequency range corresponding to the unstable region for the zero-damping case.

### 3.3. Force in the fruit stem

#### 3.3.1. Stem is perpendicular to excitation direction.

**Fig 10:**
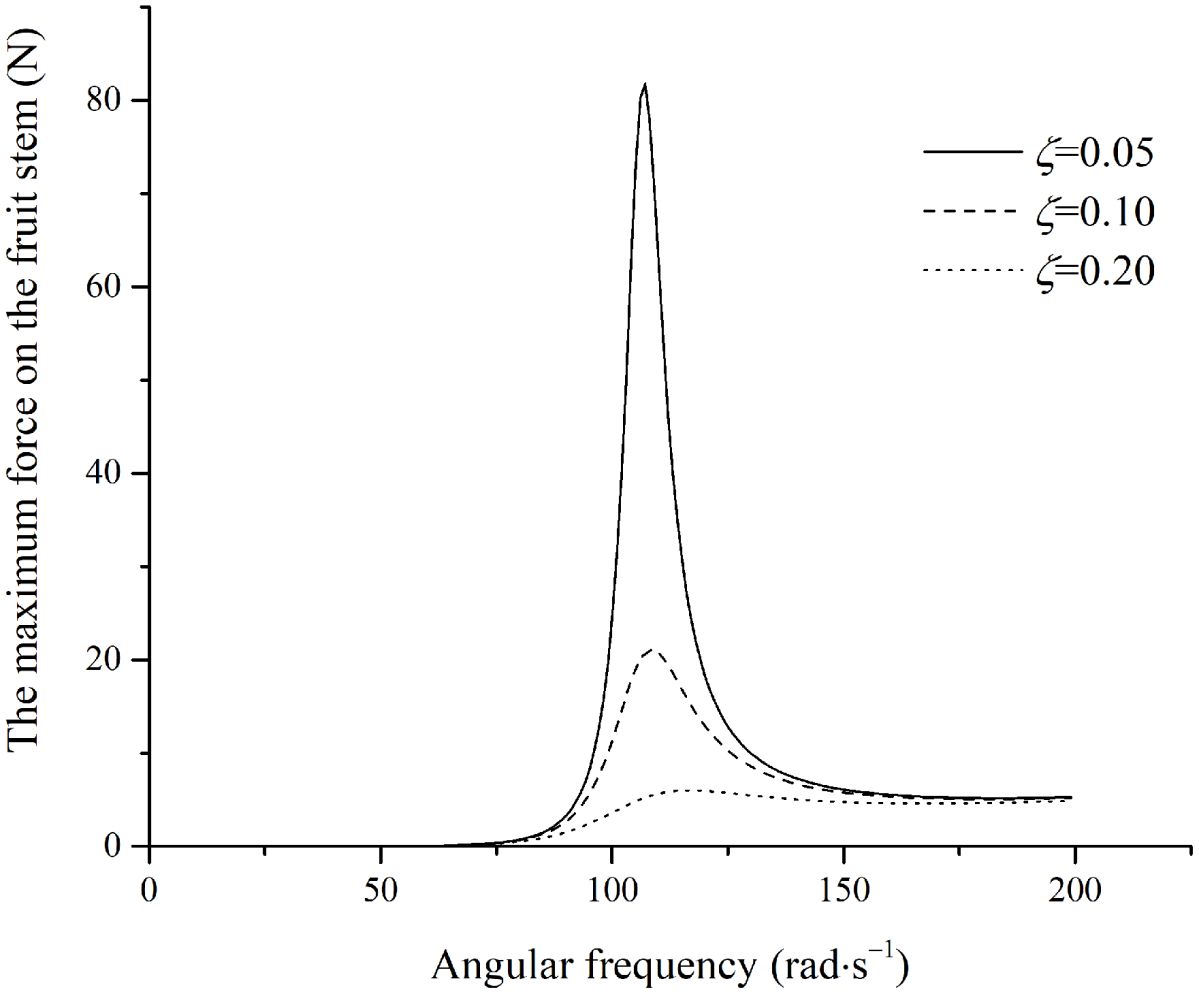
Maximum force in the stem under perpendicular excitation.

Fig 10 presents the simulation curves depicting the force in the fruit stem during the detachment process under different excitation frequencies.

In Fig 10, the influence of the fruit weight is negligible. When the excitation frequency approaches the natural frequency, the force in the fruit stem reaches a peak value. Subsequently, as the excitation frequency increases further, the force in the stem decreases. At very high frequencies, the force in the fruit stem asymptotically approaches $mg+{\left(\frac{maA_{s}\omega }{J}\right)}^{\text{2}}$. Under these conditions, the force in the stem becomes independent of the damping ratio and is primarily governed by the excitation frequency.

According to Table 1, the maximum tensile strength of the fruit stem, is $\left[\sigma \right]\cdot \frac{\pi d^{\text{2}}}{\text{4}}\text{=}7.79\text{N}$. In Fig 10, for smaller damping ratios, the excitation frequency must exceed approximately $80\text{rad}\cdot {\text{s}}^{-1}$ for the tensile force in the fruit stem to surpass its maximum tensile strength, thereby detaching the fruit. This required excitation frequency is greater than the frequency associated with the instability of the fruit stem.

#### 3.3.2. Stem is parallel to excitation direction.

**Fig 11:**
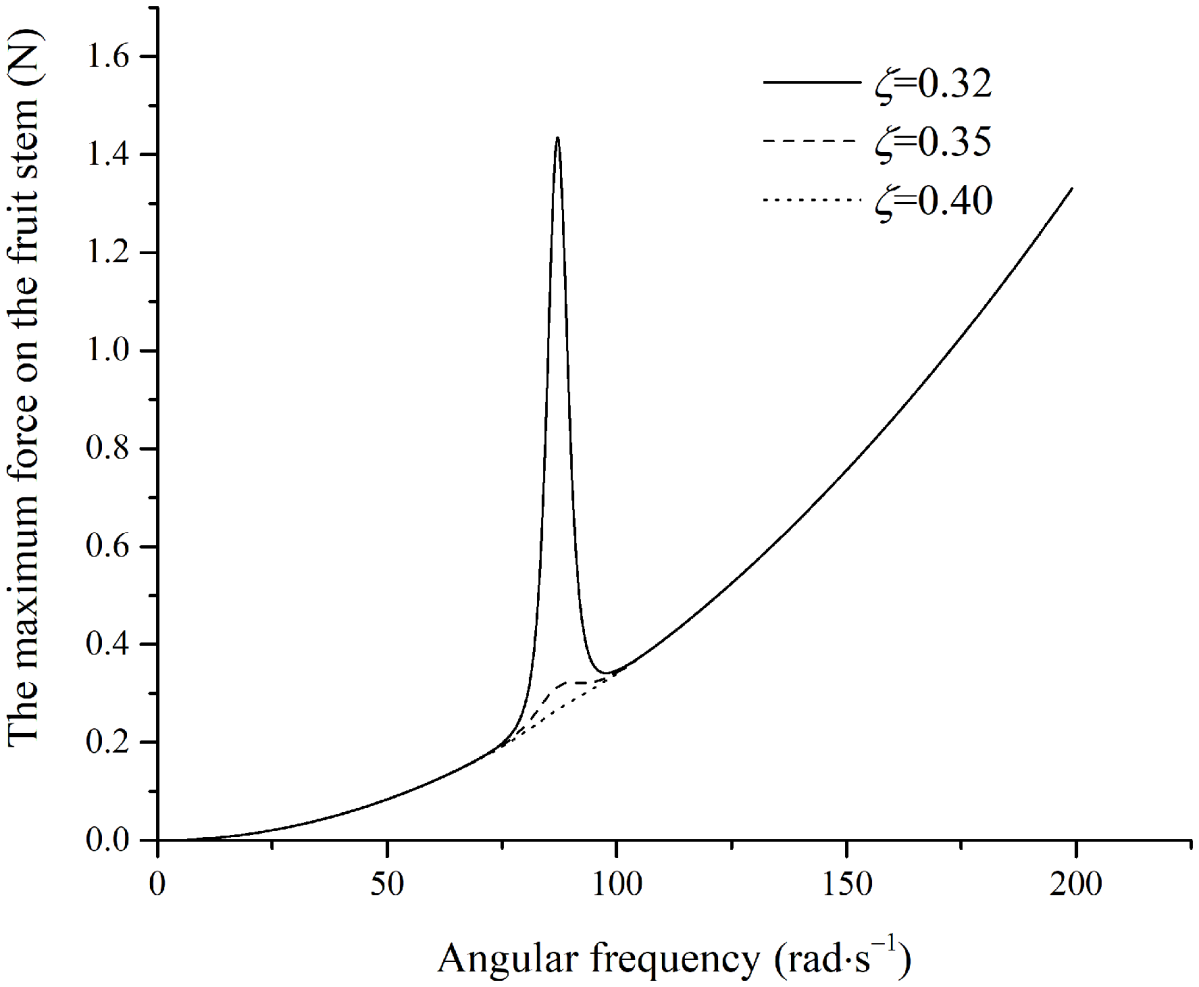
Maximum force in the stem under parallel excitation.

The relationship between the force in the fruit stem and the excitation frequency was plotted for selected damping ratios of 0.32, 0.35, and 0.4, as shown in Fig 11.

In Fig 11, the maximum force on the fruit stem is consistently less than the maximum tensile strength of the fruit stem, indicating that fruit detachment does not occur under these conditions. Detachment occurs only at a low damping ratio when the stem vibration enters the unstable region, where the maximum tensile force exceeds the stem’s tensile strength.

### 3.4. Moment in the fruit stem

#### 3.4.1. Stem is perpendicular to the excitation direction.

**Fig 12:**
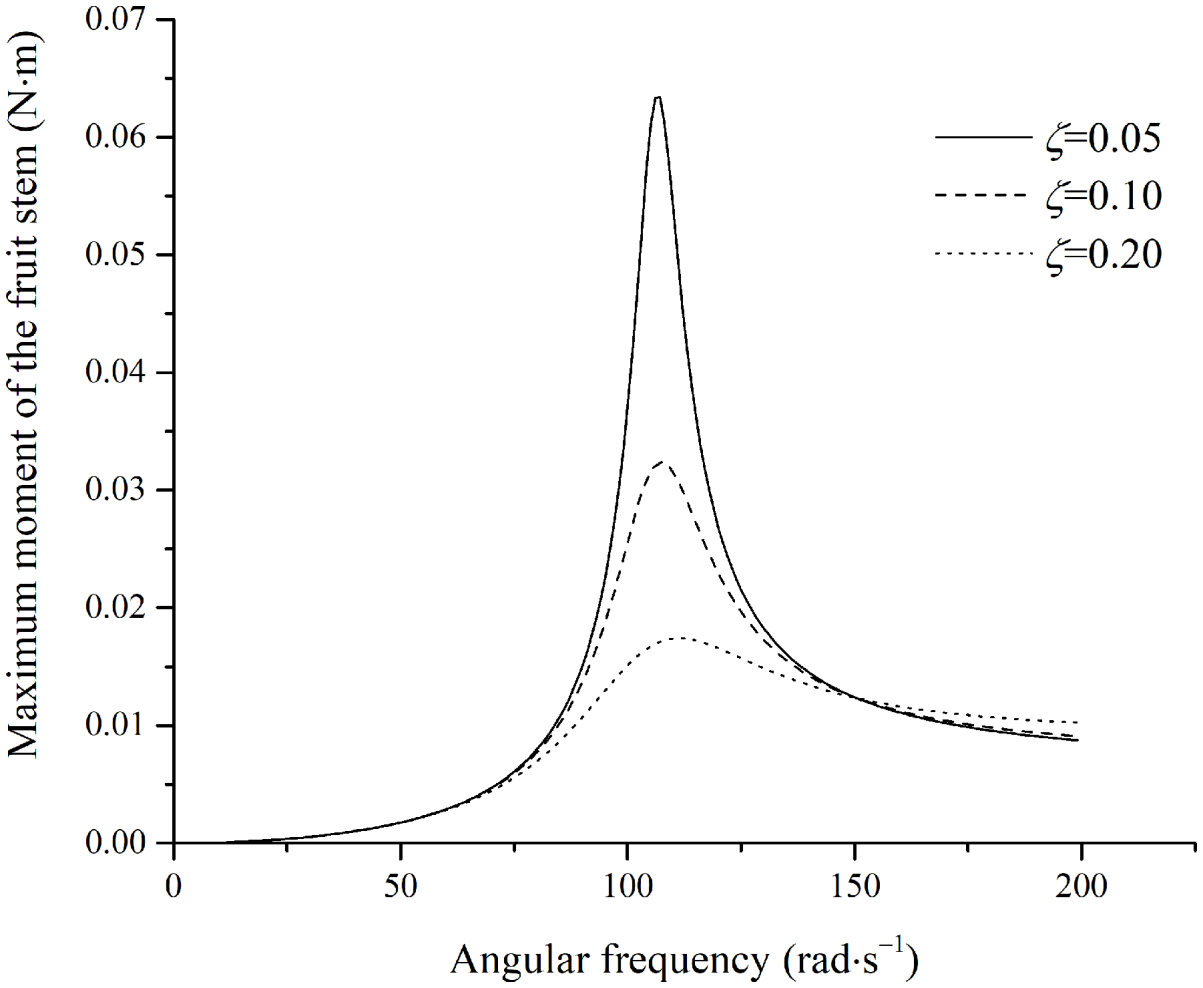
Maximum moment in the stem under perpendicular excitation.

The simulated moment in the fruit stem is shown in Fig 12.

As shown in Fig 12, the moment initially increases and then decreases. Subsequently, with increasing frequency, the moment approaches $\frac{maA_{s}\mu \omega }{J}$. When the frequency is sufficiently high, a larger damping ratio results in a greater moment. According to the data in Table 1, the maximum allowable moment for the fruit stem is $M=\frac{\left[\sigma \right]\pi d^{\text{3}}}{32}\approx 0.00052\text{N}\cdot \text{m}$. Once the excitation frequency reaches 30.1rad/s, the curves for all damping ratios become essentially identical, and the maximum moment on the fruit stem reaches 0.000537N·m. This value exceeds the maximum allowable moment in the fruit stem, resulting in fruit detachment.

#### 3.4.2. Stem is parallel to excitation direction.

**Fig 13:**
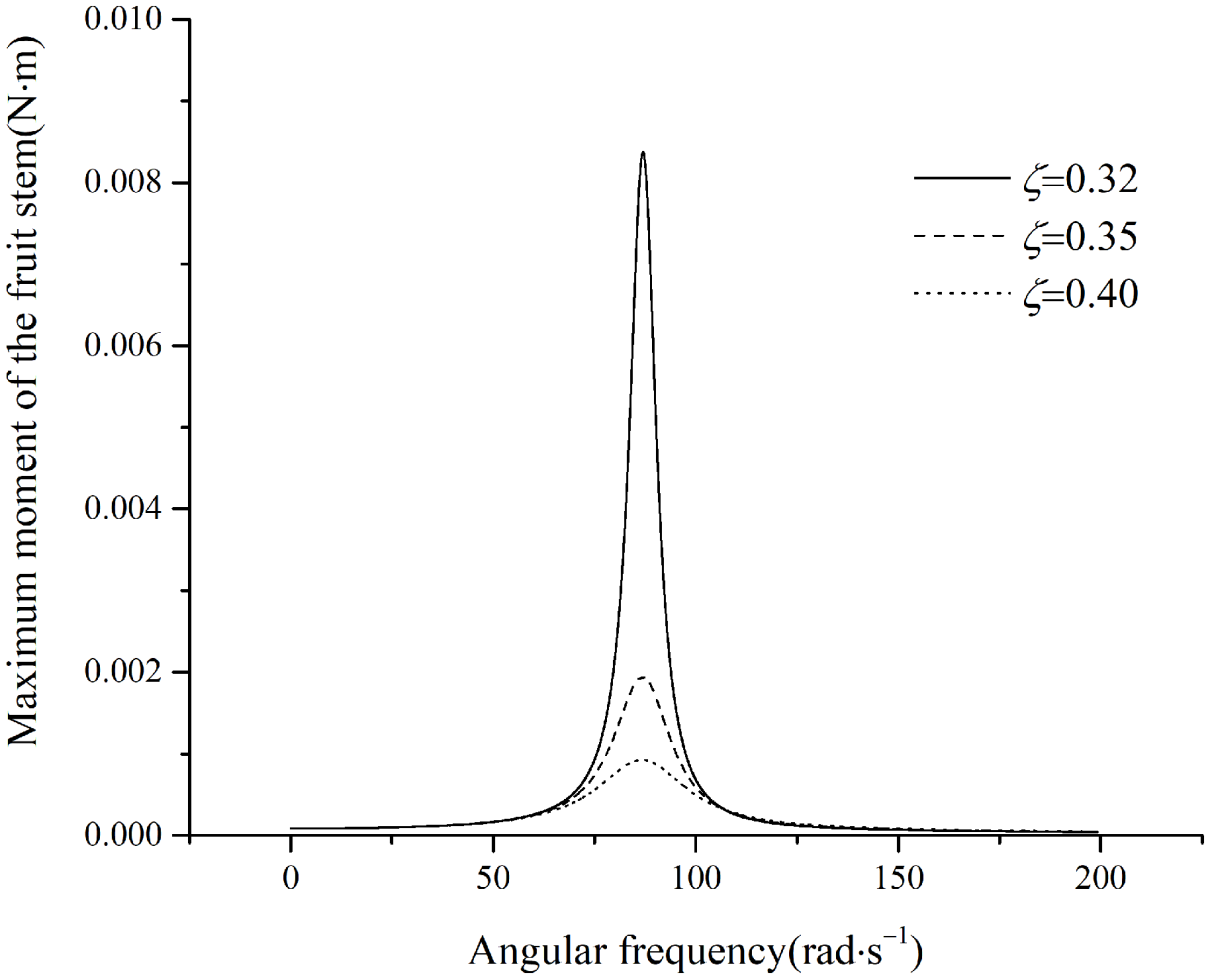
Maximum moment in the stem under parallel excitation.

The simulated moment in the fruit stem is shown in Fig 13.

In Fig 13, for a damping ratio of 0.40, the moment in the fruit stem at an excitation frequency of $75.2\text{rad}\cdot {\text{s}}^{-\text{1}}$ exceeds the maximum allowable moment 0.00052N·m. This frequency lies within the range of unstable stem vibration, which occurs at smaller damping ratios. Since fruit stems typically exhibit very low damping ratios, the excitation frequencies used for removing fruits that are obscured by foliage or that have relatively higher damping often fall within this unstable region for the stem. Therefore, it can be concluded that fruit detachment is caused by unstable vibration of the fruit stem.

If the angle between the fruit stem and the excitation direction lies between perpendicular and parallel, the maximum moment in the fruit stem will fall between the moments generated in the perpendicular and parallel configurations. The excitation frequency required for fruit detachment ranges from $\text{30.1rad}\cdot {\text{s}}^{-\text{1}}$ to the boundary of the unstable vibrational region.

### 3.5. Finite element analysis of stem vibration

Finite element analysis was performed to numerically investigate the vibration of the fruit stem. Blue Honeysuckle fruit exhibits an irregular morphology, and the attachment orientation of the fruit stem on the branch varies. To simplify the analysis, a three-dimensional model of the fruit stem and fruit was developed in ABAQUS, in which the fruit was idealized as an ellipsoid, to determine the relationship between vibration amplitude and excitation frequency, as well as the stress distribution within the fruit stem.

The fruit and stem were assigned their respective mechanical parameters as listed in Table 1. In the theoretical analysis, the mass of the fruit stem was neglected. For the finite element analysis, the density of the fruit stem was set to that of the branch. Since the fruit was modeled as an ellipsoid, its density was set to ${\text{1530kg/m}}^{\text{3}}$ base on the mass value provided in Table 1. Both the fruit and stem were modeled as linearly elastic materials. The same damping ratio was assumed for the stem and the fruit. The model was meshed, with a refined mesh applied to the fruit stem region to improve accuracy.

**Fig 14:**
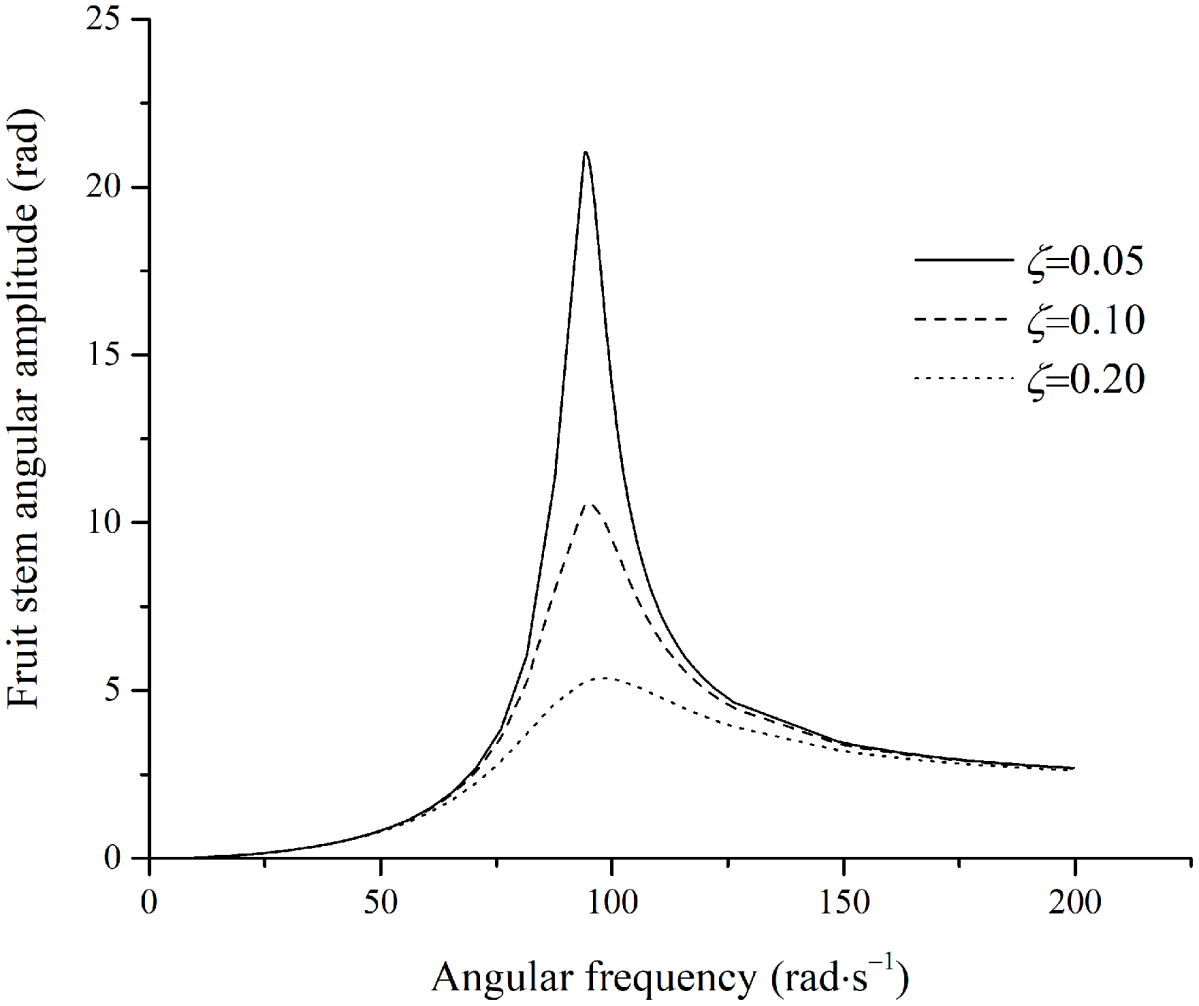
Simulated angular amplitude of the stem under perpendicular excitation.

An excitation force simulating the harvesting excitation was applied at the root of the fruit stem. Modal analysis was performed first to extract the fundamental natural frequency, followed by a frequency sweep analysis. When the fruit stem was perpendicular to the excitation direction, the numerical amplitude of the fruit stem versus the excitation frequency is presented in Fig 14.

**Fig 15:**
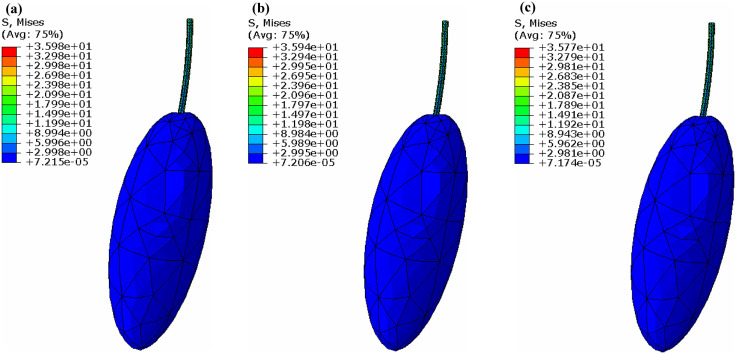
von Mises stress distribution in the stem and fruit under perpendicular excitation. (a) ζ=0.05 (b) ζ=0.1 (c) ζ=0.2.

The resulting amplitude of the fruit stem is very close to the theoretical values plotted in Fig 6. The amplitude of the fruit stem approaches a constant value as the excitation frequency increases, which is consistent with the theoretical analysis. At a frequency of $\text{24.23rad}\cdot {\text{s}}^{-\text{1}}$, the von Mises stress distribution in the fruit stem and fruit obtained from the finite element analysis is shown in Fig 15.

The maximum stress consistently occurs at the root of the fruit stem. For a damping ratio of 0.05, the von Mises stress is 35.98MPa, which exceeds the maximum allowable stress for the fruit stem 35.30MPa. The excitation frequency is close to the theoretical value of 30.1rad/s. The maximum stresses at this excitation frequency for damping ratios of 0.1 and 0.2 also show little difference.

**Fig 16:**
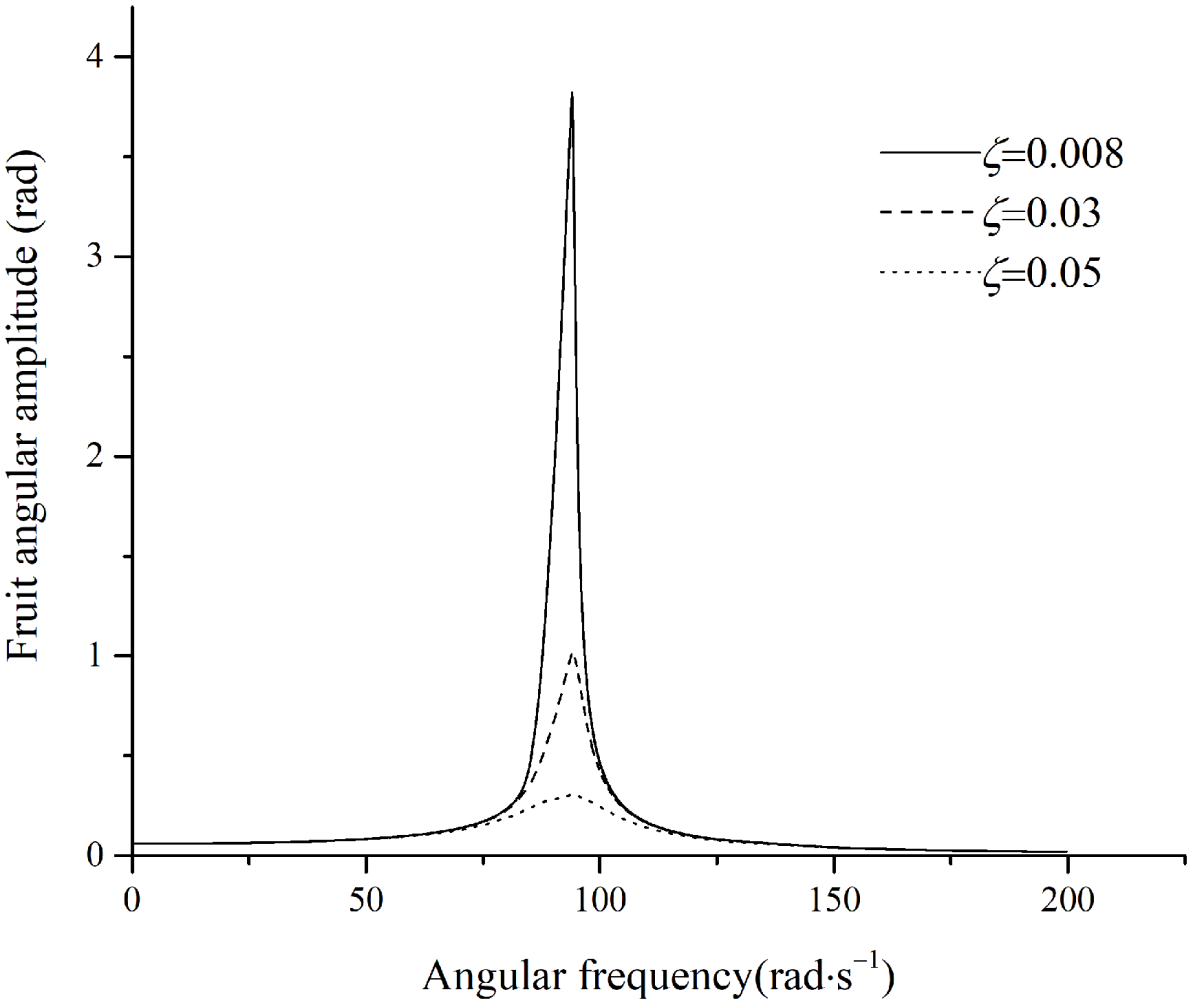
Simulated angular amplitude of the stem under parallel excitation.

When the fruit stem is parallel to the excitation direction, the computed amplitude of the fruit stem is shown in Fig 16.

**Fig 17:**
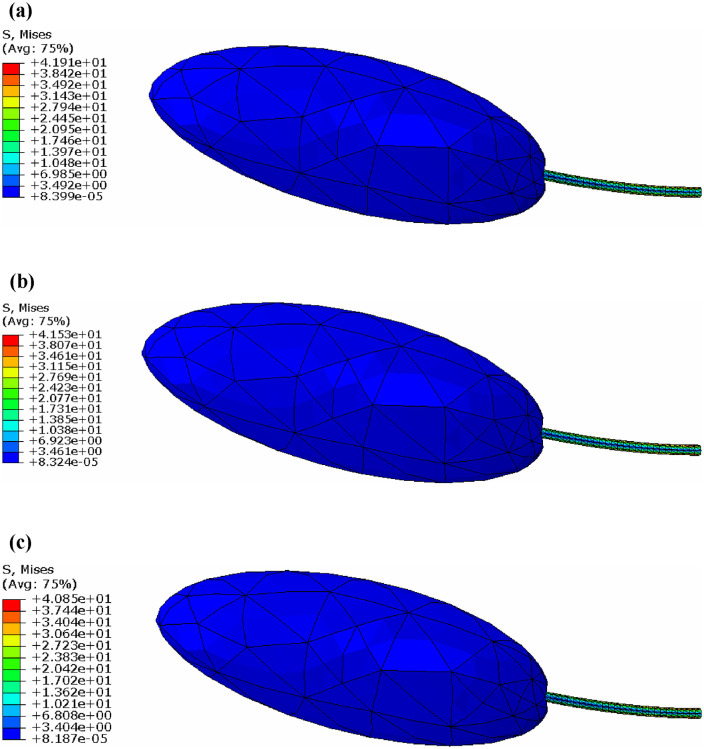
von Mises stress distribution in the fruit stem and fruit under parallel excitation. (a) ζ=0.32 (b) ζ=0.35 (c) ζ=0.40.

The output amplitude of the fruit stem approaches zero as the excitation frequency increases to a very high value, aligning with the theoretical findings. The amplitude curve closely matches the theoretical results plotted in Fig 9. At a frequency of $70.52\text{rad}\cdot {\text{s}}^{-\text{1}}$, the von Mises stress distribution in the fruit stem and fruit obtained from the finite element analysis is shown in Fig 17.

The maximum stress is observed at the root of the fruit stem. For a damping ratio of 0.40, the von Mises stress in the fruit stem is 40.85MPa, exceeding its maximum allowable stress of 35.30MPa. This frequency is very close to the theoretical excitation frequency of $75.2\text{rad}\cdot {\text{s}}^{-\text{1}}$ mentioned earlier. This also indicates that for a fruit stem with a high damping ratio, the excitation frequency required to detach the fruit falls within the instability region associated with fruit stems having a lower damping ratio.

### 3.6. Field experiment results

**Fig 18:**
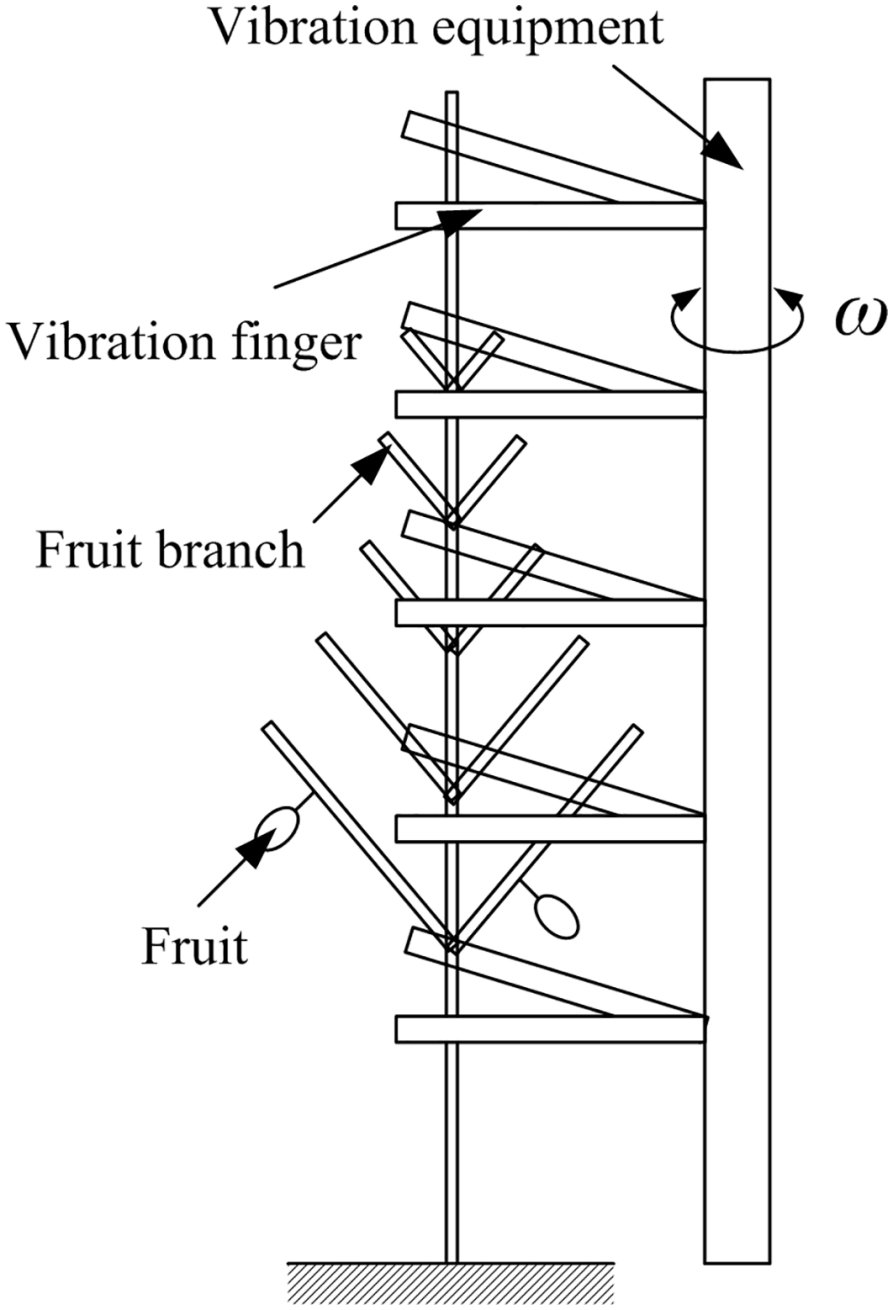
Schematic of the vibration-based harvesting device.

The harvesting efficiency of Blue Honeysuckle is influenced by numerous factors, including excitation frequency, amplitude, excitation duration, crop variety, and climatic conditions. In this experiment, the excitation frequency and amplitude were selected as the variables for investigating berry harvesting. The excitation frequency corresponds to the rotational speed of the drive motor, while the amplitude represents the displacement of the vibrating device acting on the tree. The experimental setup is shown in Fig 18.

Two rows of harvesting fingers reciprocate to vibrate the Blue Honeysuckle trees. This arrangement allows the assumption that the amplitude and frequency of vibration remain uniform throughout the tree. The harvesting efficiency is determined primarily by measuring the mass of harvested fruits and is calculated as:


$$k_{\text{1}}=\frac{m_{\text{1}}}{m_{\text{1}}+m_{\text{2}}}\times 100$$ (51)

where $m_{\text{1}}$ is the mass of detached fruits, and $m_{\text{2}}$ is the mass of fruits remaining attached to the branches. The damage ratio mainly includes fruit damage caused by the vibration excitation and is expressed as:


$$k_{\text{2}}=\frac{m_{\text{3}}}{m_{\text{1}}}\times 100$$ (52)

where $m_{\text{3}}$ is the mass of fruits damaged during harvesting.

#### 3.6.1. Vibrational harvesting of fruit trees under natural conditions.

The experimental subjects were naturally-fruiting fruit trees aged 5–8 years. The trees were subjected to vibrational harvesting without any pretreatment. The harvesting efficiency and damage ratio are as presented in the Table 2.

**Table 2:** The experimental results of Blue Honeysuckle harvesting.

Test No.	Excitation Frequency ($\text{rad}\cdot {\text{s}}^{-\text{1}}$)	Excitation Amplitude (mm)	Harvesting Efficiency	Damage Ratio
1	31.42	39	43.24	1.43
2	31.42	53	60.48	4.17
3	41.87	39	59.92	2.28
4	41.87	53	70.37	5.05
5	52.33	39	72.78	3.32
6	52.33	53	96.11	6.56
7	62.83	39	84.45	4.54
8	62.83	53	100	9.21
9	73.27	39	99.73	5.32
10	73.27	53	100	11.68

The experimental data indicate that fruit detachment is correlated with rotational speed and amplitude. Elevated rotational speeds and larger amplitudes lead to a higher harvest efficiency. This is because, for fruits with stems oriented at an angle to the excitation direction, increased rotational speed and amplitude more readily induce instability in the stem’s vibration, leading to detachment. For fruits with stems perpendicular to the excitation direction, the increase in rotational speed and amplitude increases the bending moment, as described by Eqs.(46) and(48), thereby promoting fruit detachment. At the same rotational speed, the experimental data indicate that an increase in amplitude more readily causes fruit damage. This is because fruit damage is primarily associated with the fruit’s velocity. During vibratory harvesting, the maximum velocity of the fruit can be expressed as $v_{max}=A_{s}\omega$. Neglecting the vibration of the fruit stem and assuming the force required to detach the fruit $mA_{s}\omega^{\text{2}}$ is a constant, then under the same detachment force condition, a larger amplitude corresponds to a higher fruit velocity, thereby increasing the risk of fruit damage. Therefore, vibratory harvesting should aim to increase the frequency while reducing the amplitude.

When the excitation frequency reaches $\;31\mathrm{.42}\text{rad}\cdot {\text{s}}^{-\text{1}}$, a significant number of fruits begin to detach. The experimental onset frequency is very close to the theoretical excitation frequency of $\;30\mathrm{.1}\text{rad}\cdot {\text{s}}^{-\text{1}}$ for stems perpendicular to the excitation direction. When the excitation frequency reaches $73.27\text{rad}\cdot {\text{s}}^{-\text{1}}$, nearly all fruits detach. This observation aligns closely with the previously mentioned critical frequency of $70.9\text{rad}\cdot {\text{s}}^{-\text{1}}$ for an amplitude of 39mm and a damping ratio of zero, at which instability occurs for vibrations with stems parallel to the excitation direction. When the excitation frequency was varied from $\;31\mathrm{.42}\text{rad}\cdot {\text{s}}^{-\text{1}}$ to $73.27\text{rad}\cdot {\text{s}}^{-\text{1}}$, fruits with stem orientations transitioning from perpendicular to parallel relative to the excitation direction began to detach progressively, and the harvesting efficiency increased steadily.

#### 3.6.2. Vibration harvesting of fruits with manually selected stem orientations.

For naturally grown fruit trees, a comparative harvesting experiment was conducted by selectively removing fruits with stems oriented in specific directions, in order to verify the effect of pedicel orientation on fruit detachment. Since the experimental excitation direction was approximately horizontal, fruits with horizontal stems aligned with the excitation direction, while those with vertical stems were approximately perpendicular to it.

**Fig 19:**
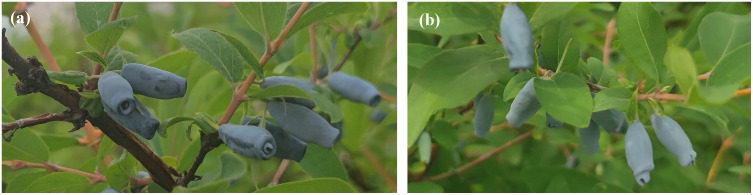
Stem distribution on branches under different orientations. (a) Angle between fruit stem and horizontal direction less than π/4. (b) Angle between fruit stem and vertical direction less than π/4. Note: This is an original figure created by the authors.

In the test group, fruits with stems forming a relatively small angle with the vertical direction were manually removed from the trees. Only fruits whose stems formed an angle of less than π/4 with the horizontal direction were retained, resulting in stems being approximately parallel to the excitation direction. The fruit distribution on the plant after this manual removal is shown in Fig 19 (a). Subsequently, vibration harvesting was performed on the entire tree. The harvesting efficiency and damage ratio of the fruits from this experiment are presented in the Table 3. As a control group, fruits with stems forming a relatively large angle with the vertical direction were manually removed. Only fruits whose stems formed an angle of less than π/4 with the vertical direction were retained, resulting in stems being approximately perpendicular to the excitation direction. The fruit distribution after this manual removal is shown in Fig 19 (b). Vibration harvesting was then conducted on the entire tree. The corresponding harvesting efficiency and damage ratio are presented in the Table 4.

**Table 3:** The experimental results of approximate horizontal stem harvesting.

Test No.	Excitation Frequency ($\text{rad}\cdot {\text{s}}^{-\text{1}}$)	Excitation Amplitude (mm)	Harvesting Efficiency	Damage Ratio
1	31.42	39	35.18	1.65
2	41.87	39	52.51	2.98
3	52.33	39	65.43	3.96
4	62.83	39	78.87	5.38
5	73.27	39	99.54	5.82

**Table 4:** The experimental results of approximate vertical stem harvesting.

Test No.	Excitation Frequency ($\text{rad}\cdot {\text{s}}^{-\text{1}}$)	Excitation Amplitude (mm)	Harvesting Efficiency	Damage Ratio
1	31.42	39	51.46	1.05
2	41.87	39	66.72	2.14
3	52.33	39	79.64	3.26
4	62.83	39	90.95	4.57
5	73.27	39	100	5.45

As shown in Table 3, since the theoretical analysis was conducted under the assumption of steady-state vibration without considering the transient vibration caused by instantaneous impact on the fruit branches, a considerable number of fruits were still detached at lower excitation frequencies. As the frequency increased, the fruit stem entered a state of unstable vibration, leading to fruit detachment. Due to the artificial removal of fruits with vertically oriented stems, the harvesting efficiency was significantly reduced compared with that in Table 2, indicating that fruit detachment is strongly correlated with the orientation angle of the fruit stem. The variation in damage rate remained largely consistent with that in Table 2.

Similarly, Table 4 shows that transient impact-induced vibration of the branches resulted in fruits detachment at relatively low excitation frequencies. Because the fruit stem formed a small angle with the vertical direction, the fruit harvesting efficiency was higher than that in Table 3 under all frequency conditions, and the difference between the two was substantial. The experiments were conducted under identical conditions except for the orientation of the fruit stems, yet this single factor led to a notable difference in harvesting efficiency. This further confirms that fruit detachment is closely related to the angle of the fruit stem.

## 4. Discussion

The four fundamental modes of fruit motion include flapping, pendulum motion, rotation, and torsion. Although the theoretical model considered only the pendulum motion of the fruit-stem system, the results show good agreement with field experiments. Due to the relatively thin stems and comparatively heavy fruit of Blue Honeysuckle, its motion closely resembles that of a simple pendulum. The experiments demonstrate that pendulum motion is the dominant mode, while flapping, rotation, and torsion are of secondary importance.

The orientations of Blue Honeysuckle stems and the excitation direction are predominantly between vertical and parallel. This indicates that fruit detachment occurs under both stable and unstable vibration conditions. Since the unstable vibration frequency of the fruit-stem system occurs when the branch amplitude is 39mm with a frequency of $70.9\text{rad}\cdot {\text{s}}^{-1}$, experiments confirm that most fruits had already detached at frequencies lower than this threshold. This suggests that fruit detachment under stable vibration is dominant, which can be attributed to the fact that most stems are oriented nearly vertically.

In little cases, the fruit stems are predominantly curved. When manual removing the fruit, alignment should be based on the long axis direction of the fruit. Since many fruits are closely clustered together, after a specific fruit is removed, the stems of adjacent fruits may undergo positional changes. If these altered stems no longer meet the predefined criteria, they must also be removed. For experiments where the angle between the fruit stem and the horizontal direction is less than π/4, even after meticulous inspection, a very small number of stems may still exhibit an angle greater than π/4 during the actual experiment due to various factors. However, this does not affect the validity of the experimental conclusions. For experiments where the angle between the fruit stem and the vertical direction is less than π/4, the influence of gravity ensures that, after selective fruit removal, cases where the stem forms an angle greater than π/4 with the vertical direction are essentially non-occurring.

Fruits in the upper canopy exhibit larger oscillation amplitudes and detach more readily, whereas fruits in the lower canopy remain attached. The harvesting apparatus in the vibration experiments was positioned approximately 300mm above the ground, and observations revealed that numerous fruits below this height failed to detach.

In both the theoretical and finite element analyses, all physical parameters used in simulations were based on average values. The fruits that remained attached during experiments were predominantly green or unripe. This is because the maximum allowable detachment force for unripe Blue Honeysuckle stems is higher than that for mature fruits. As indicated by the experimental results, both the maturity of Blue Honeysuckle and their canopy position significantly influence the efficacy of fruit detachment.

Although the effect of instantaneous impact on fruit detachment was not investigated in this study, this omission does not undermine the findings regarding the influence of the angle between the stem and the excitation direction on harvesting efficiency, as well as the physical conditions for the detachment mechanisms, such as stress failure and vibrational instability. These findings provide a theoretical foundation for the design of harvesting equipment for Blue Honeysuckle.

## 5. Conclusions

This study developed a dynamic model that incorporates the angle between the fruit stem and the excitation direction, which clarifies the physical mechanisms and key governing factors of fruit detachment during the vibratory harvesting of Blue Honeysuckle. Theoretical analysis demonstrated that the initial orientation angle of stem significantly affects the excitation frequency required for fruit detachment, as well as the detachment characteristics. When the stem is perpendicular to the excitation direction, fruit detachment occurs because the maximum bending stress exceeds the allowable stress of the stem. Conversely, when the stem is parallel to the excitation direction, the system enters vibrational instability, leading to detachment as the vibration amplitude approaches infinity.

Finite element analysis of the fruit-stem system showed close agreement with theoretical predictions, Specifically, when the stem is perpendicular to the excitation direction, the von Mises stress in the stem exceeds its maximum allowable stress at a relatively low excitation frequency. In contrast, when the stem is parallel, the stress surpasses the allowable stress only within the frequency range corresponding to the instability region. Field experiments further validate the reliability of this theoretical framework: as the excitation frequency increases from $\;31\mathrm{.42}\text{rad}\cdot {\text{s}}^{-\text{1}}$ to $73.27\text{rad}\cdot {\text{s}}^{-\text{1}}$, the harvesting efficiency improved from partial to nearly complete, a trend consistent with the theoretically predicted trend. A comparative experiment involving manual selection of stem orientation directly confirmed the significant impact of stem orientation on fruit detachment.

This study establishes the critical role of the angle between the fruit stem and the excitation direction in the dynamics of vibratory harvesting. It defines the physical conditions and transition boundaries between the two detachment mechanisms: stress failure and vibrational instability, providing a quantitative theoretical basis for the design of harvesting equipment for Blue Honeysuckle and other delicate berry crops that balances efficiency with low damage. The proposed modeling and analytical methodology also provide a general research framework for agricultural engineering problems involving the dynamic separation of flexible biomaterials.

## Supporting information

S1 File Parameter. Basic parameters of Blue Honeysuckle used in the mathematical model.
(XLSX)

S2 File Experimental. Experimental data from Blue **Honeysuckle harvesting.**
(XLSX)
